# Defect-Energy-Targeted Lattice Repair Delivers High Thermoelectric Performance in Magnesium Antimonide

**DOI:** 10.1021/jacs.6c02279

**Published:** 2026-03-14

**Authors:** Jiahao Jiang, Minhui Yuan, Yuntian Fu, Yanqi Huang, Wenjie Li, Jingyi Lyu, Zeqing Hu, Shenghua Liu, Ran He, Yanglong Hou, Jing Shuai

**Affiliations:** 1 School of Materials, Shenzhen Campus of Sun Yat-sen University, Shenzhen 518107, China; 2 Leibniz Institute for Solid State and Materials Research IFW Dresden, Dresden 01069, Germany; 3 State Key Laboratory for Modification of Chemical Fibers and Polymer, Materials & College of Materials Science and Engineering, 12475 Donghua University, Shanghai 201620, China; 4 State Key Laboratory of Optoelectronic Materials and Technologies, 26469 Sun Yat-Sen University, Guangzhou 510275, China

## Abstract

Magnesium-based Mg_3_(Sb,Bi)_2_ has emerged as a premier candidate for waste-heat recovery. However, its performance is fundamentally capped by intrinsic Mg vacancies that severely scatter carriers. Here, we overcome this bottleneck via a defect-energy-targeted lattice repair strategy, substituting labile Mg sites with homologous alkaline-earth metals (Ca, Sr, Ba). Theoretical calculations reveal that the lower electronegativity of these dopants strengthens the local metal–Sb bonding, drastically raising the vacancy formation energy from ∼0.97 to ∼2.42 eV. This thermodynamic stabilization effectively “repairs” the lattice, suppressing vacancy generation and yielding a ∼35% boost in carrier mobility without compromising carrier concentration. Simultaneously, the heavy dopants induce mass fluctuations and strain fields that, coupled with dense dislocations, minimize the lattice thermal conductivity to ∼0.4 W m^–1^ K^–1^ at 773 K. The synergy of restored charge transport and suppressed heat propagation leads to a record-high figure of merit (*zT*) of ∼2.1 at 773 K and an outstanding average *zT* of ∼1.5 in Mg_3.2_Ba_0.005_Sb_1.5_Bi_0.49_Te_0.01_. Remarkably, a single-leg device demonstrates a conversion efficiency of ∼14%, outperforming state-of-the-art n-type thermoelectrics. This work demonstrates that targeting defect energetics is a powerful, broadly applicable approach to breaking the performance ceilings of Zintl-phase thermoelectrics.

## Graphical abstract

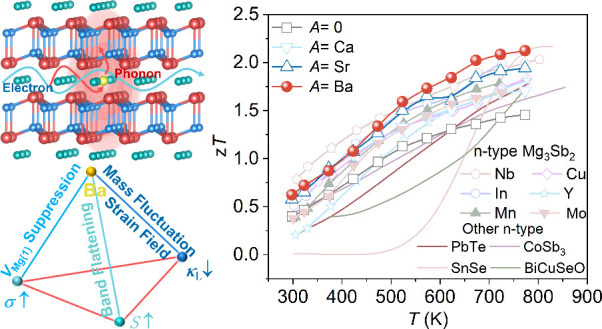

## 1 Introduction

Thermoelectric devices represent a transformative solution for addressing global energy challenges by utilizing the Seebeck and Peltier effects to achieve solid-state, emission-free heat-to-electricity conversion.^1,2^ Optimizing materials for the medium-temperature range (300–800 K) is particularly crucial, as this regime encompasses over 60% of industrial waste heat.^3,4^ However, widespread application remains hindered by the limited conversion efficiency of current thermoelectric materials, governed by the dimensionless figure of merit *zT* (= *S* ^2^σ*T*/κ). The inherent trade-offs between the Seebeck coefficient (*S*), electrical conductivity (σ), and thermal conductivity (κ) pose a persistent challenge.^5^ Despite decades of research yielding laboratory breakthroughs, commercial applications are still dominated by bismuth telluride (Bi_2_Te_3_)-based materials.^6^ This reliance on tellurium, characterized by extremely low crustal abundance (Te < 0.001 ppm) and mediocre mechanical properties (fracture toughness: ∼1 MPa·m^1/2^), underscores both the significant challenges and immense opportunities in developing next-generation thermoelectric alternatives.^7,8^

Magnesium-based thermoelectrics, particularly n-type Mg_3_(Sb,Bi)_2_, have emerged as compelling candidates due to their eco-friendly composition, lightweight nature, and promising performance.^9^ Initial p-type behavior caused by Mg vacancies was overcome by synthesizing with excess Mg, enabling materials like Mg_3.2_Sb_1.5_Bi_0.49_Te_0.01_ to achieve remarkable *zT* values of ∼1.5 at ∼750 K.^10–12^ This performance stems from synergistic advantages: high conduction band valley degeneracy (*N*_v_ = 6) for excellent electronic transport, mass contrast between Sb/Bi for phonon scattering,^10^ and strong anharmonicity suppressing lattice thermal conductivity (κ_lat_).^13^ Consequently, n-type Mg_3_(Sb,Bi)_2_-based devices demonstrate efficiencies rivaling commercial Bi_2_Te_3,_ ^14,15^ alongside superior mechanical properties (fracture toughness > 3 MPa·m^1/2^) and abundant raw materials,^8,16^ positioning them as viable, sustainable alternatives for large-scale waste heat recovery.

Nevertheless, unlocking the full potential of n-type Mg_3_(Sb,Bi)_2_ requires overcoming severe transport challenges.^17^ Extensive efforts have focused on optimizing carrier concentration through heterovalent doping (e.g., transition metals,^18–20^ lanthanides,^21–23^ and group III/VI elements^24,25^ ). Concurrently, grain boundary engineering—via elevated sintering temperatures,^26–28^ novel sintering aids (e.g., Mg_2_Cu),^29^ or reduced grain boundary barriers (e.g., Nb incorporation)^30^—has proven critical to mitigate detrimental interfacial states arising from Mg deficiency and lattice mismatch, leading to notable *zT* improvements.

However, despite these advances, the system remains fundamentally limited by strong carrier scattering from intrinsic Mg(1) vacancies.^31,32^ These defects, stemming from the high volatility of Mg and its low vacancy formation energy, persist even under Mg-overstoichiometric conditions,^33^ acting as stubborn scattering centers that cap the carrier mobility. Crucially, while grain boundary engineering addresses mesoscale transport, it fails to resolve this atomic-scale bottleneck. Historical precedents in state-of-the-art thermoelectrics underscore that direct defect suppression via bond strengthening is transformative: for instance, sulfur doping in n-PbTe effectively suppresses Pb vacancies by enhancing chemical bonding,^34^ and Cu incorporation in p-SnSe eliminates Sn vacancies to enable multiband convergence.^35^ Drawing inspiration from these breakthroughs, it becomes evident that a strategy targeting the thermodynamic root of defect formation—specifically by increasing the energy barrier for vacancy generation—is essential for transcending the current performance ceilings of magnesium antimonide.

Herein, we implement a defect-energy-targeted lattice repair strategy to fundamentally resolve the vacancy bottleneck in n-type Mg_3_(Sb,Bi)_2_. As illustrated in Figure 1a, this strategy is designed to decouple electron and phonon transport: by substituting labile Mg sites with homologous alkaline-earth metals (Ca, Sr, Ba), we aim to thermodynamically stabilize the cation sublattice while simultaneously introducing phonon scattering centers. Our density functional theory (DFT) calculations validate that the lower electronegativity of these heavy group-II dopants enhances the local metal–Sb bonding, drastically elevating the Mg-vacancy formation energy from ∼0.97 eV to ∼2.42 eV. This “lattice repair” effectively suppresses intrinsic defects, yielding a ∼35% recovery in carrier mobility. Synergistically, the heavy dopants induce mass fluctuations and strain fields that, coupled with dense dislocations, minimize the lattice thermal conductivity to ∼0.4 W m^–1^ K^–1^. Consequently, the optimized Mg_3.2_Ba_0.005_Sb_1.5_Bi_0.49_Te_0.01_ achieves a record-high peak *zT* of ∼2.1 and an average *zT* of ∼1.5, outperforming most state-of-the-art n-type thermoelectrics (Figure 1b). Finally, a fabricated single-leg device demonstrates a conversion efficiency of ∼14% at a temperature difference of 473 K (Figure 1c), establishing our lattice-repair strategy as a definitive route to commercially viable, high-efficiency power generation.

**Figure 1:**
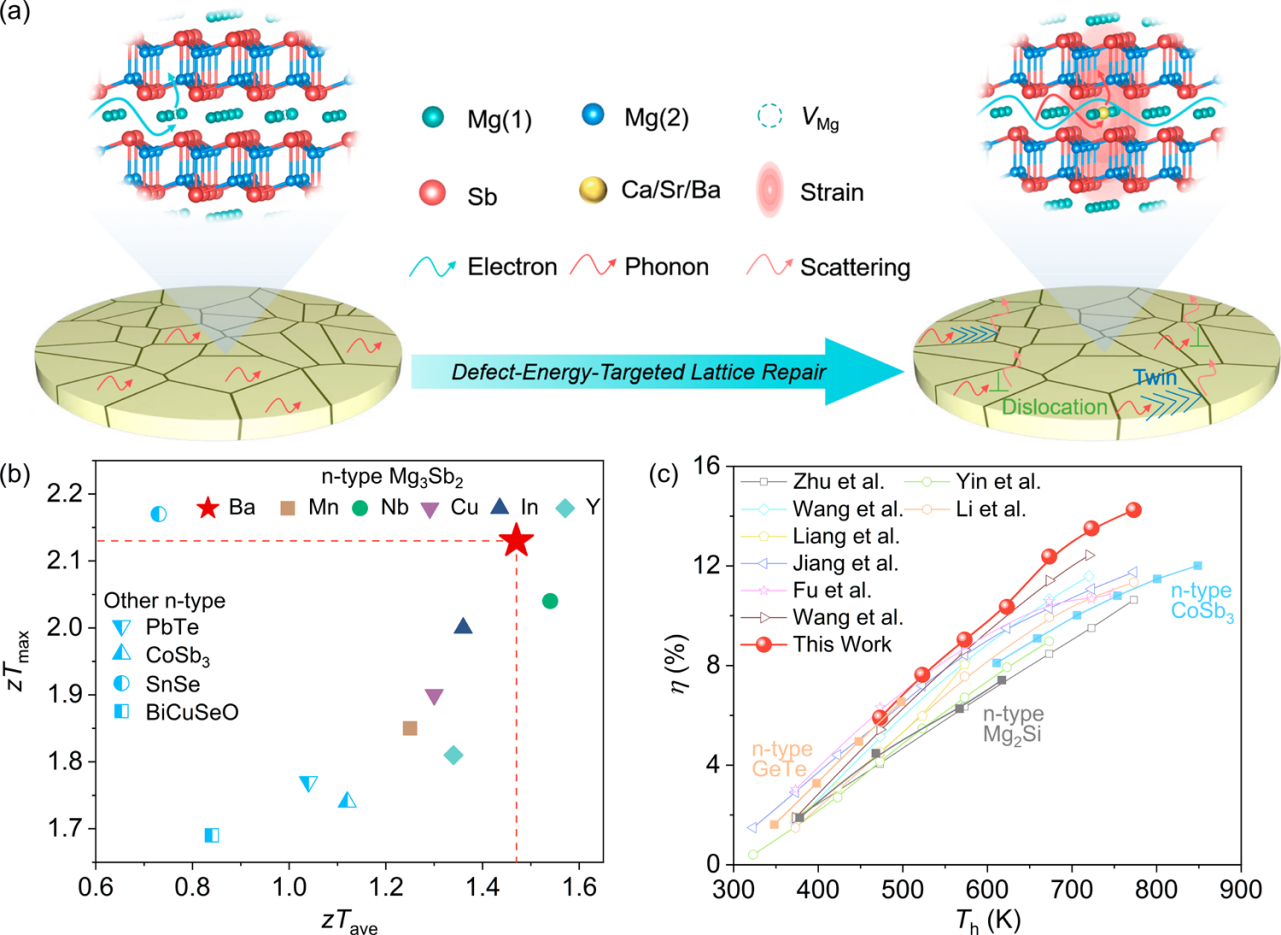
Enhanced thermoelectric (TE) performance and power generation efficiency via defect-energy-targeted lattice repair strategy. (a) Schematic diagram of the differential regulation of electron and phonon transport enabled by the defect-energy-targeted lattice repair strategy utilizing Ca/Sr/Ba dopants. (b) Comparison of the maximum TE figure of merit (*zT*_max_) and average *zT* (*zT*_avg_) between the n-type Mg_3_(Sb,Bi)_2_ systems developed in this work and other reported n-type TE materials.^19,20,24,30,34,36–39^ (c) Conversion efficiency of the fabricated module compared with other state-of-the-art modules.^18,24,33,40–47^

## 2 Result and Discussion

### 2.1 Thermodynamic Lattice Repair and Defect Suppression

To rationalize the efficacy of the defect-energy-targeted lattice repair strategy, we first investigated the thermodynamic stability of the cation sublattice using density functional theory (DFT) calculations. In the pristine Mg_3_(Sb,Bi)_2_ lattice, the loosely bonded Mg(1) sites are prone to vacancy formation, creating a plethora of scattering centers that degrade carrier mobility. We hypothesized that substituting these labile sites with homologous alkaline-earth elements (Ca, Sr, Ba) would reinforce the local chemical environment due to their significantly lower electronegativity compared to Mg (Mg: 1.31 > Ca: 1.00 > Sr: 0.95 > Ba: 0.89).

Our calculations reveal a striking correlation between the dopant species and defect energetics. As shown in Figure 2a, the incorporation of alkaline-earth metals substantially increases the formation energy of the Mg(1) vacancy (*V*_Mg_). Among them, Ba induces the most profound stabilization: the *V*_Mg_ formation energy surges from ∼0.97 eV in the pristine lattice to an almost insurmountable ∼2.42 eV in the Ba-repaired system. This >150% increase effectively suppresses the thermodynamic driver for vacancy proliferation. To elucidate the atomic-scale origin of this stabilization, we analyzed the electron localization function (ELF) and crystal orbital Hamilton population (COHP). The ELF maps (Figure 2b) display a significant accumulation of charge density between Ba and Sb/Bi atoms, indicative of enhanced ionic–covalent bonding. Concurrently, the −COHP analysis (Figure 2c) further reveals the electronic origin of this enhancement. Specifically, the black curve represents the Mg(1)–Sb interaction in the pristine sample, while the green curve corresponds to the Ba–Sb interaction in the Ba-doped sample. The significantly higher −COHP value of the Ba–Sb bond near the Fermi level confirms that Ba introduction strengthens the local bond network.

**Figure 2:**
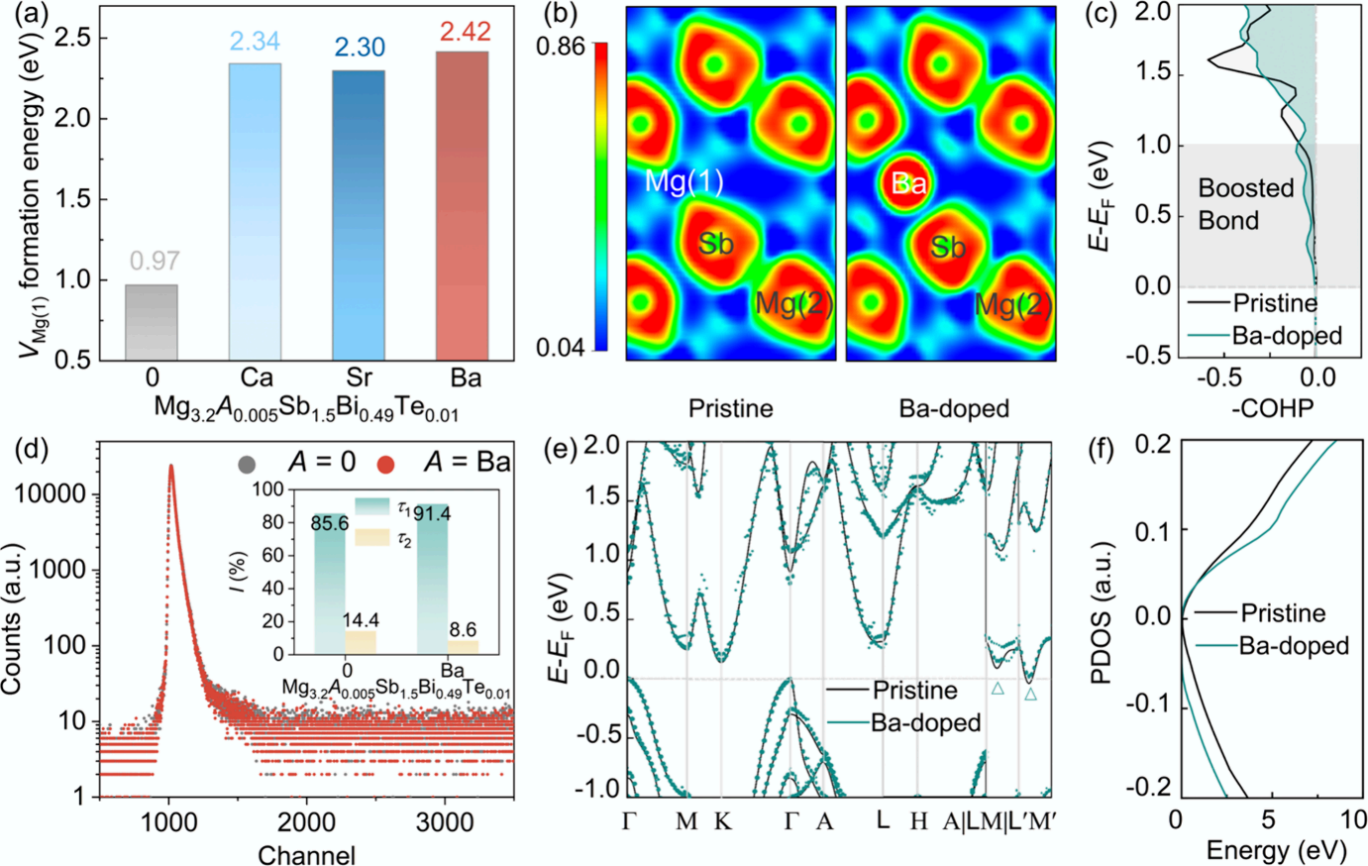
Experimental characterization and theoretical calculation of electrical transport properties for Mg_3.2_ *A*_0.005_Sb_1.5_Bi_0.49_Te_0.01_ (*A* = 0, Ca, Sr, Ba) samples. (a) Calculated formation energies of Mg(1) vacancies. (b) The electron localization function (ELF) of the pristine and Ba-doped samples along the (112̅0) plane. (c) Negative crystal orbital Hamilton population (−COHP) analysis comparing the Mg(1)–Sb interaction in pristine Mg_54_Sb_36_ (black) and the Ba–Sb interaction in BaMg_53_Sb_36_ (green). (d) Positron annihilation lifetime spectra (PALS) of *A* = 0 and *A* = Ba samples; the inset shows the relative intensities of positron lifetimes τ_1_ and τ_2_ derived from PALS analysis. (e) Electronic band structures of Mg_54_Sb_36_ and BaMg_53_Sb_36_. (f) Partial density of states (PDOS) for *A* = 0 and *A* = Ba samples.

To experimentally validate this “lattice repair” mechanism and confirm dopant incorporation, we synthesized Mg_3.2_ *A*_0.005_Sb_1.5_Bi_0.49_Te_0.01_ (*A* = Ca, Sr, Ba) samples. X-ray diffraction (XRD) patterns (Figure S1) reveal that all samples crystallize in the single-phase anti-α-La_2_O_3_ structure (space group *P*3̅*m*1).^10^ Rietveld refinement results demonstrate that the unit cell volume exhibits a monotonic expansion with increasing doped ion radius (Figure S2), consistent with Vegard’s law.^48^ This behavior indicates successful incorporation of dopants into the lattice sites, which is further confirmed by transmission electron microscopy (TEM) structural characterization presented in Section 2.3 (Figure 5).

Crucially, we employed positron annihilation lifetime spectroscopy (PALS) to directly probe the vacancy evolution, as shown in Figure 2d. The spectra were resolved into a bulk lifetime (τ_1_) and a defect-related lifetime (τ_2_), with the intensity *I* proportional to the defect concentration (Table S1). For the Ba-doped sample, the intensity *I* decreases significantly compared to the pristine counterpart, experimentally confirming the suppression of cation vacancies predicted by our DFT results. Interestingly, the τ_2_ value itself increases, exceeding the characteristic lifetime of single Mg vacancies (∼254 ps).^49^ This suggests that while the number of point defects (vacancies) is reduced, the nature of the remaining defects shifts toward larger-sized clusters or extended defects (e.g., dislocations or twins), a feature that will be further discussed in the context of thermal conductivity (Section 2.3). Consistent with PALS, Rietveld refinement of the XRD data also indicates a higher occupancy of Mg sites (i.e., fewer vacancies) in the doped samples (Tables S2 and S3).

Beyond structural stabilization, the introduction of Ba also reconstructs the electronic structure. The lattice strain and chemical potential shift result in an upward shift of the conduction band minimum (CBM) and a widening of the bandgap (Figure 2e). Accompanying this shift is a flattening of the dispersion near the CBM, which leads to a redistribution of the density of states (DOS) and an increased carrier effective mass (Figure 2f). This electronic evolution, while potentially affecting mobility, is a trade-off that is compensated by the drastic reduction in impurity scattering, as will be demonstrated in the transport property analysis.

### 2.2 Restored Carrier Mobility and Electrical Performance

Building upon the thermodynamic stabilization of the cation sublattice, we evaluated the impact of lattice repair on the electrical transport properties. As shown in Figure 3a, the electrical conductivity (σ) of all Group IIA-doped samples is significantly enhanced across the entire temperature range (300–773 K). Hall effect measurements (Figure 3b and Figure S3) reveal that this enhancement is primarily driven by a systematic recovery in carrier mobility (μ), rather than a drastic change in carrier concentration (*n*). Specifically, at 300 K, μ surges from ∼90 cm^2^ V^–1^ s^–1^ in the pristine sample to ∼120 cm^2^ V^–1^ s^–1^ in the Ba-doped counterpart—a ∼35% improvement.

**Figure 3:**
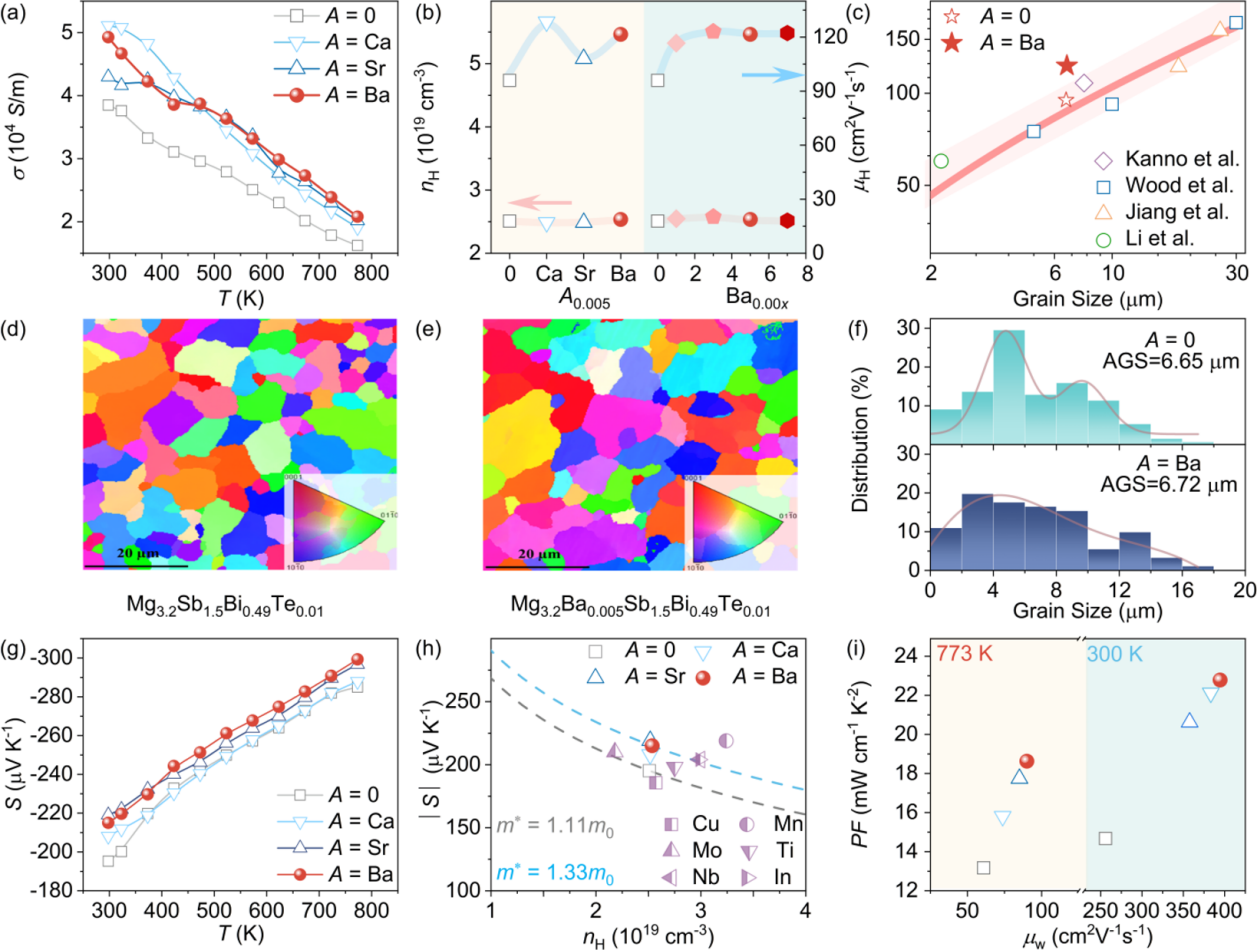
Electrical transport properties of Mg_3.2_ *A*_0.005_Sb_1.5_Bi_0.49_Te_0.01_ (*A* = 0, Ca, Sr, Ba) samples. (a) Temperature dependence of the electrical conductivity (σ). (b) The carrier concentration (*n*) and carrier mobility (μ), including additional data for Mg_3.2_Ba_0.00*x*_Sb_1.5_Bi_0.49_Te_0.01_ (*x* = 1, 3, 5, 7) samples. (c) Correlation between carrier mobility (μ) and grain size, compared with reported Mg_3_(Sb,Bi)_2_ systems possessing similar chemical compositions and carrier concentrations.^18,26,28,30^ Electron backscatter diffraction (EBSD) crystal-orientation maps for (d) *A* = 0 and (e) *A* = Ba samples. (f) Grain size distributions derived from EBSD analysis for *A* = 0 and *A* = Ba samples. (g) Temperature dependence of the Seebeck coefficient (*S*). (h) Pisarenko plot showing the *S* as a function of carrier concentration. Gray and light blue lines represent single parabolic band (SPB) model predictions with effective masses m* = 1.11*m*_0_ and 1.33 *m*_0_, respectively, compared with literature data.^18,20,24,30,36,42^ (i) Power factor (*PF*) and weighted mobility (μ_w_) at 300 and 773 K.

This mobility restoration directly validates our theoretical hypothesis in Section 2.1. In conventional n-type Mg_3_(Sb,Bi)_2_, ionized Mg vacancies act as potent scattering centers that severely limit electron transport. By thermodynamically elevating the vacancy formation energy via Ba substitution, we have effectively suppressed these scattering centers at the atomic level. To rule out extrinsic microstructural contributions, we analyzed the grain size statistics using electron backscatter diffraction (EBSD) (Figure 3d–f). Contrary to typical grain boundary engineering strategies where mobility gains track with grain growth, our Ba-doped samples exhibit a stable grain size (∼6.7 μm), virtually identical to the pristine sample (Figure 3c).^18,26,28,30^ This decoupling of mobility from grain size serves as compelling evidence that the transport enhancement originates from the purification of the intratelluric lattice—specifically, the reduction of intrinsic point defects—rather than mesoscale interface modifications.

Interestingly, the carrier concentration remains optimized at ∼2.5 × 10^19^ cm^–3^ for all samples (Table S4). This stability arises from a beneficial self-compensation mechanism: while the suppression of acceptor-like Mg vacancies tends to increase the electron concentration, the simultaneous widening of the bandgap (predicted in Figure 2e) suppresses intrinsic thermal excitation. This delicate balance ensures that the material retains an optimal doping level without requiring complex counter-doping adjustments.

Beyond mobility, the lattice repair strategy also reshapes the electronic density of states (DOS), leading to a synergistic enhancement in the Seebeck coefficient (*S*). As shown in Figure 3g, all samples display n-type behavior, with Group IIA doping yielding higher |*S*| values compared to the pristine baseline. Applying the Single Parabolic Band (SPB) model (Figure 3h), we calculated the density-of-states effective mass (m*). Notably, Ba incorporation increases m* from 1.11*m*_0_ to 1.33*m*_0_. This experimental finding aligns perfectly with the DFT-predicted band flattening near the conduction band minimum (Section 2.1, Figure 2f). Unlike traditional dopants (e.g., Cu, Mo, Ti) that often degrade mobility when increasing mass,^18,20,42^ our strategy achieves a rare “win-win” scenario: the reduced scattering (higher μ) compensates for the heavier mass, while the flattened bands (higher m*) boost the Seebeck coefficient.

Consequently, the simultaneous optimization of μ and m* leads to a remarkable improvement in the power factor (*PF* = *S* ^2^σ), as shown in Figure S4. The Ba-doped sample achieves a peak PF of ∼23 μW cm^–1^ K^–2^ at 300 K (∼64% increase) and maintains ∼19 μW cm^–1^ K^–2^ at 773 K (Figure 3i). Further analysis using the weighted mobility (μ_w_ ≈ μ­(m*/*m*_e_)^3/2^) confirms that Ba doping yields the most pronounced enhancement among all alkaline-earth elements (Figure S5).^50^ Even at ultralow doping levels (0.1 at. %), electrical transport properties uplift is evident (Figure 3b and Figures S6 and S7), underscoring the universal and potent efficacy of this thermodynamic defect-engineering approach.

### 2.3 Microstructure Evolution and Synergistic Phonon Scattering

We systematically evaluated the thermal transport properties to understand the impact of the lattice repair strategy on phonon propagation. While alkaline-earth doping substantially enhances electrical conductivity—thereby increasing the electronic thermal conductivity (κ_e_ = *L*σ*T*)^51^—the total thermal conductivity (κ_tot_ = κ_e_+ κ_lat_) remains suppressed (Figure 4a). This decoupling indicates a dramatic reduction in the lattice component (κ_lat_). As shown in Figure 4b, Ba doping yields the most pronounced effect, achieving an ultralow κ_lat_ of ∼0.4 W m^–1^ K^–1^ at 773 K. This trend can be rationalized by the dopant size effect: while all alkaline-earth dopants (Ca, Sr, Ba) suppress intrinsic vacancies via the same bonding mechanism, the extent of secondary extended defects scales with ionic radius mismatch. Ba, having the largest mismatch with Mg, induces the strongest lattice strain, thereby promoting the highest density of dislocations and nanotwins compared to Ca and Sr. Notably, this suppression is evident even at minute doping levels (0.1 at. %) and saturates at ∼0.5 at. %, underscoring the high potency of Ba as a phonon scatterer (Figure 4b and S6). Crucially, when plotting κ_lat_ against grain size (Figure 4c),^18,30,42,52–54^ the Ba-doped samples defy the conventional trend where larger grains typically lead to higher κ_lat_. Instead, they maintain ultralow thermal conductivity despite having grain sizes comparable to the pristine material, suggesting that the phonon scattering mechanism has shifted from grain boundary domination to intrinsic lattice interactions.

**Figure 4:**
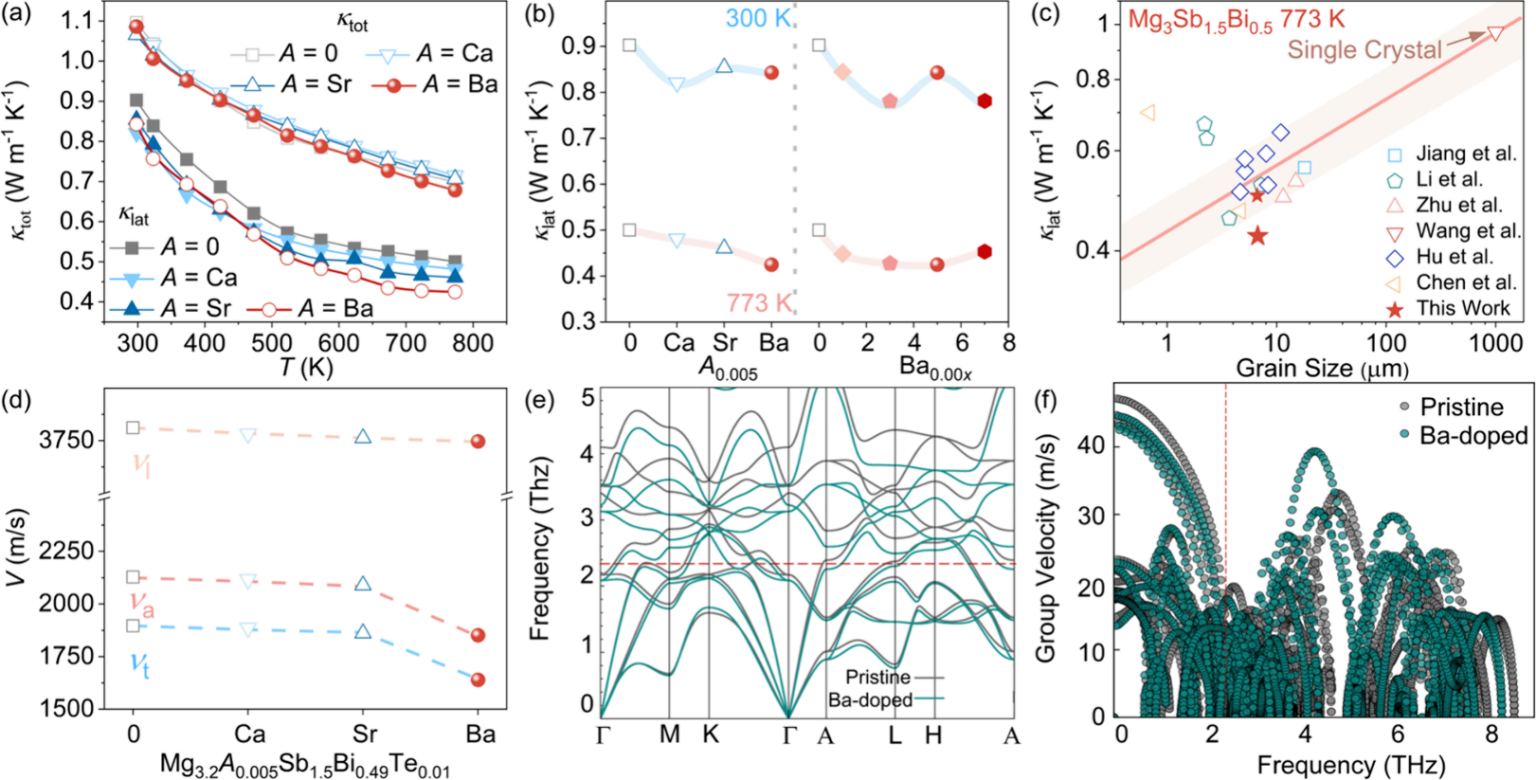
Thermal transport properties and phonon dynamics analysis. (a) Temperature dependence of total thermal conductivity (κ_tot_) and lattice thermal conductivity (κ_lat_) for Mg_3.2_ *A*_0.005_Sb_1.5_Bi_0.49_Te_0.01_ (*A* = 0, Ca, Sr, Ba) samples. (b) κ_lat_ at 300 and 700 K for Mg_3.2_ *A*_0.005_Sb_1.5_Bi_0.49_Te_0.01_ samples and Mg_3.2_Ba_0.00*x*_Sb_1.5_Bi_0.49_Te_0.01_ (*x* = 0, 1, 3, 5, 7) samples. (c) Correlation between κ_lat_ and grain size compared with reported Mg_3_(Sb,Bi)_2_ systems of similar chemical compositions.^18,30,42,52–54^ (d) Longitudinal (*v*_l_), transverse (*v*_t_), and average (*v*_a_) sound velocities. (e) Phonon dispersion curves and (f) group velocities for Mg_54_Sb_36_ and BaMg_53_Sb_36_.

To elucidate the microscopic origin of this reduction, we analyzed the phonon group velocity (*v*_g_) and dispersion relations. According to the kinetic theory $\kappa \mathrm{lat}=\frac{1}{3}c_{v}v_{g}^{2}\tau$ , (where *c*_v_ is the specific heat and τ is the phonon relaxation time^55^), the reduction in *v*_g_ directly suppresses heat transport. Sound velocity measurements (Figure 4d) reveal a monotonic decrease in average sound velocity with increasing dopant atomic mass. Specifically, the heavy Ba atoms not only introduce significant mass contrast but also induce lattice distortion that increases the c/a ratio (Figure S2). Phonon spectrum calculations (Figure 4e) demonstrate that this distortion triggers a significant softening of low-frequency optical modes (<2 THz), particularly in the transverse acoustic branches. This softening flattens the phonon dispersion, resulting in a substantial reduction in group velocity across the low-frequency spectrum (Figure 4f). Thus, the synergistic combination of mass-fluctuation scattering and lattice softening creates a formidable barrier to heat propagation.

To visualize the structural defects responsible for this scattering, we employed aberration-corrected transmission electron microscopy (AC-TEM) to systematically analyze the Ba-induced microstructure. High-resolution STEM imaging along the [21̅10] zone axis reveals a pristine long-range crystalline order (Figure 5a), with sharp diffraction spots corresponding to (011̅1̅), (01̅11), and (0002) planes in the FFT pattern confirming the structural integrity of the host matrix. To precisely determine the dopant occupancy, we conducted a comparative analysis using HAADF-STEM and ABF-STEM. In the HAADF mode (Figure 5b), the low atomic number of Mg (*Z* = 12) yields minimal contrast; however, the ABF mode, which is sensitive to light elements, clearly resolves the periodic arrangement of Mg(1) atomic columns (Figure S8). This consistency with simulated structures confirms that doping does not induce phase separation. Crucially, atomic-scale HAADF-STEM analysis (Figure 5c) reveals distinct bright spots at Mg(1) sites, identifying Ba^2+^ occupation through Z-contrast. The significant ionic radius mismatch between Ba^2+^ (1.35 Å) and Mg^2+^ (0.72 Å) acts as a geometric perturbation. We quantified this perturbation using Geometric Phase Analysis (GPA) (Figure 5d). The strain maps reveal a complex local distortion field: Ba substitution generates significant negative shear strain in the ε_*xy*_ direction, corresponding to a counterclockwise lattice shear. Simultaneously, the incorporation of the large Ba ion compresses neighboring Sb atoms, introducing pronounced compressive strain in the ε_*xx*_ direction (blue regions), while weak compressive strain in the ε_δδ_ direction arises from volume compensation via the Poisson effect. This strong local distortion propagates through the rigid Mg–Sb bond network, effectively scattering high-frequency phonons.

**Figure 5:**
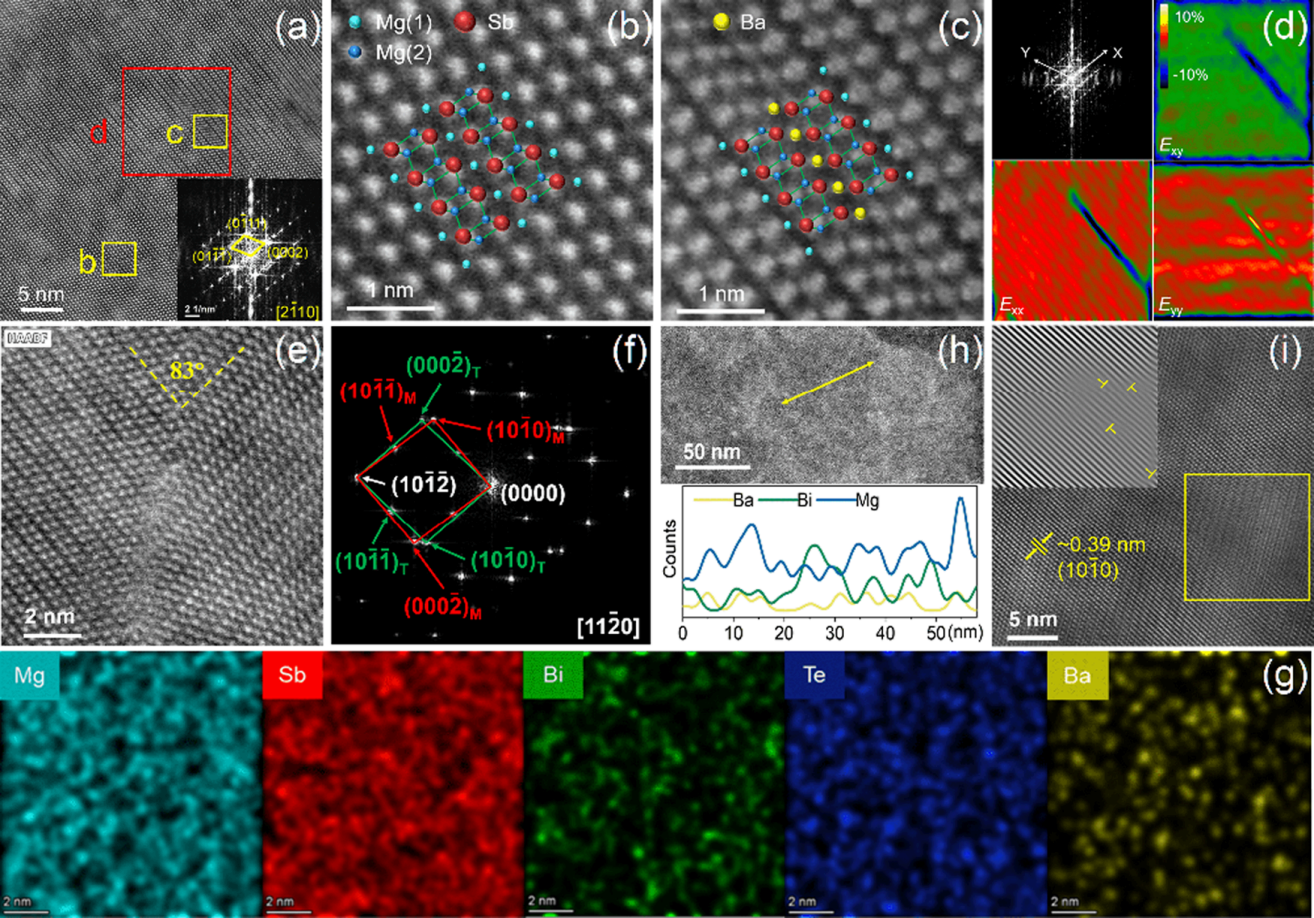
Microstructural characterization of Mg_3.2_Ba_0.005_Sb_1.5_Bi_0.49_Te_0.01_ sample. (a) High-angle annular dark-field scanning transmission electron microscopy (HAADF-STEM) image with inset showing the fast Fourier transform (FFT) pattern of the selected region. (b, c) Magnified HAADF-STEM images of the yellow boxed region from panel (a). (d) Geometric phase analysis (GPA) of the red boxed region from panel (a), displaying the strain field distribution. (e) Atomic-resolution HAADF-STEM image of the twin interface with (f) corresponding FFT pattern and (g) STEM-energy dispersive spectroscopy (EDS) elemental distribution map. (h) Low-magnification HAADF-STEM image with the yellow line segment indicating the region for EDS line scan analysis of Mg, Bi, and Ba elements. (i) HAADF-STEM image showing the (101̅0) plane with inset displaying the inverse Fast Fourier Transform (IFFT) of the yellow boxed region.

Beyond point defects, the Ba-induced lattice strain triggers the formation of extended mesoscopic defects, constructing a hierarchical scattering architecture. Atomic-resolution imaging along the [112̅0] zone axis reveals the presence of nanoscale twins with a characteristic angle of ∼83° (Figure 5e and Figure S9). FFT analysis confirms the specific crystallographic relationships: (101̅0)_M_∥(101̅0)_T_, (0002̅)_M_∥(0002̅)_T_, and (101̅2̅)_M_∥(101̅2̅)_T_. These coherent twin boundaries act as effective scattering planes for mid-to-low frequency phonons via coherent Bragg reflection.^56,57^ Crucially, despite the large ionic radius mismatch between Ba and Mg, STEM-EDS mapping (Figure 5g) reveals a homogeneous distribution of Ba throughout the matrix. We specifically examined the grain boundaries and found no evidence of Ba segregation or enrichment, confirming the formation of a solid solution rather than secondary precipitates. This uniform distribution ensures the chemical order required for electron transport while maximizing point-defect scattering. However, at a finer scale, low-magnification STEM-EDS line scanning uncovers periodic compositional fluctuations of Mg and Bi at ∼50 nm (Figure 5h and Figure S10), providing additional mass fluctuations for scattering low-frequency phonons. Finally, High-resolution TEM reveals edge dislocations on the (101̅0) plane (Figure 5i), complementing the scattering spectrum. Notably, the presence of these dense dislocations and nanotwins provides a structural basis for the increased long-lifetime component (τ_2_) observed in PALS (Figure 2d), corroborating the evolution of defects into larger clusters.

In summary, the introduction of Ba achieves a cross-scale selective structural regulation. At the atomic scale, relying on the inherent electronic structure compatibility, the system maintains perfect long-range chemical and crystallographic order to ensure high carrier mobility. Concurrently, a structurally disordered network—comprising local strain fields (ε_*xx*_ and ε_*y*__*y*_), coherent nanotwins, dislocations, and compositional fluctuations—is constructed to efficiently scatter phonons across the entire frequency spectrum. This strategy of precisely implanting disordered scattering centers within an ordered lattice successfully suppresses lattice thermal conductivity without compromising electrical performance.

### 2.4 Record-Breaking *zT* and High-Efficiency Thermoelectric Power Generation

The group-homologous lattice repair strategy culminates in a synergistic breakthrough: the simultaneous optimization of power factor (via electronic band engineering and vacancy elimination) and suppression of lattice thermal conductivity (via full-spectrum phonon scattering). To rigorously evaluate the thermoelectric figure of merit (*zT*), we employed the Dulong-Petit limit for specific heat capacity (*C*_p_) calculations. As shown in Figure S11, the Dulong-Petit values are lower than those measured by differential scanning calorimetry (DSC), ensuring that our reported *zT* values are conservative and reliable. Under this stringent benchmark, the optimized Mg_3.2_Ba_0.005_Sb_1.5_Bi_0.49_Te_0.01_ composition achieves a record-breaking peak *zT* of 2.13 at 773 K (Figure 6a). This performance not only eclipses conventional n-type benchmarks such as CoSb_3_ (*zT* ≈ 1.6) and PbTe (*zT* ≈ 1.8),^34,37^ but also rivals the intrinsically anharmonic SnSe (*zT* ≈ 2.2),^38^ establishing Mg_3_Sb_2_ as a top-tier thermoelectric candidate.

**Figure 6:**
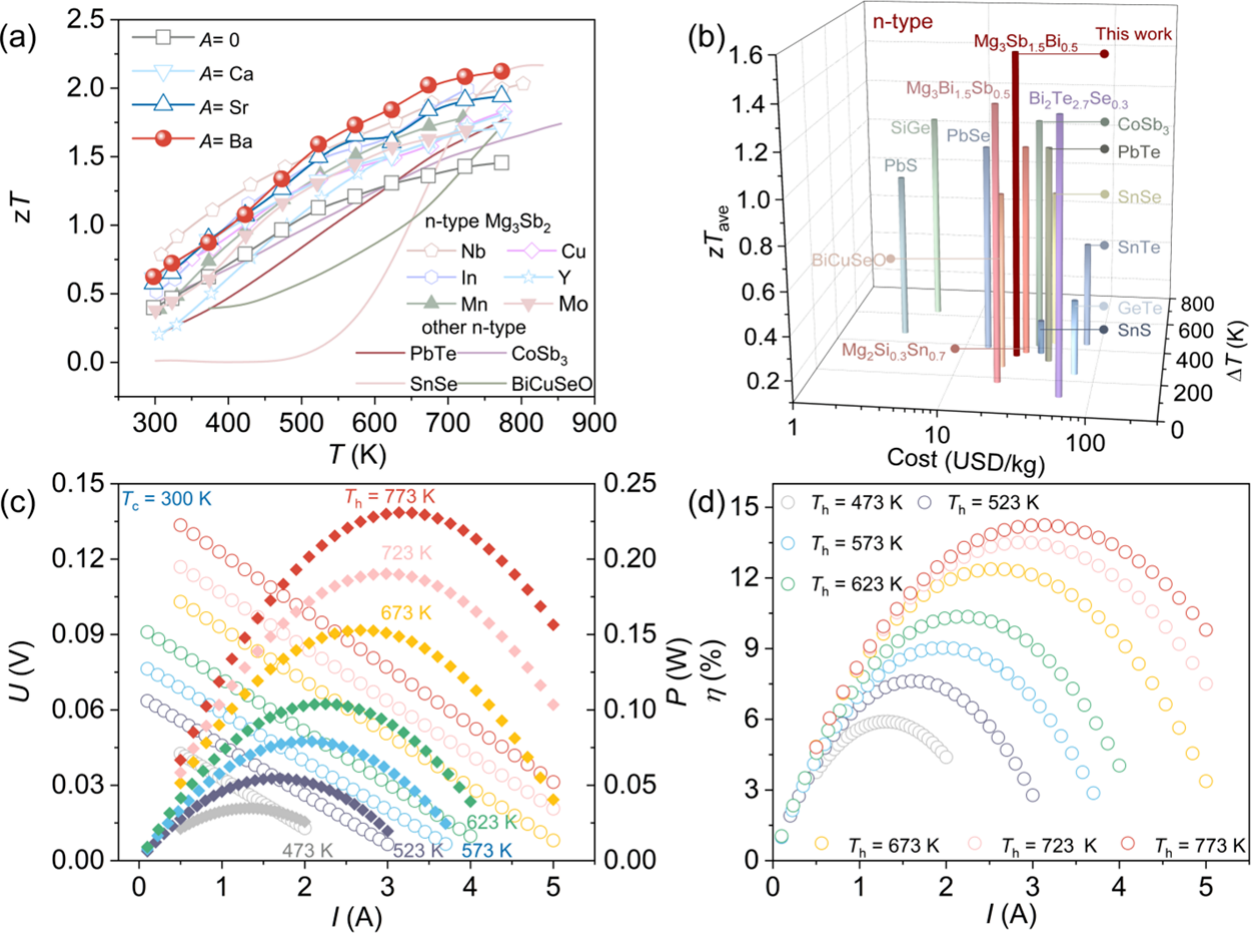
Thermoelectric performance, cost-effectiveness analysis, and device performance evaluation. (a) Temperature-dependent *zT* values of Mg_3.2_ *A*_0.005_Sb_1.5_Bi_0.49_Te_0.01_ (*A* = 0, Ca, Sr, Ba) samples compared with reported Mg_3_(Sb,Bi)_2_-based systems and other representative n-type thermoelectric materials.^19,20,24,30,34,36–39,42^ (b) Cost-performance analysis showing the relationship between material cost and average *zT* value (*zT*_avg_) for typical n-type thermoelectric materials.^34,37,39,46,58–64^ (c) Output voltage and power as a function of current for the Mg_3.2_Ba_0.005_Sb_1.5_Bi_0.49_Te_0.01_ single-leg device at various hot-side temperatures. (d) Conversion efficiency of the single-leg device as a function of temperature difference.

For practical applications, the average figure of merit (*zT*_ave_) and material cost are more critical than peak *zT*. Figure 6b presents a comprehensive comparison of mainstream n-type thermoelectric systems (Tables S5 and S6).^34,37,39,46,58–64^ Our Ba-doped material exhibits an exceptional balance, achieving a high *zT*_ave_ of ∼1.5 across the 300–773 K range while maintaining a low raw material cost of ∼$20 kg^–1^. This cost is merely ∼40% of Bi_2_Te_3_ and ∼55% of PbTe. The combination of ultrahigh efficiency and low cost positions this Mg-based system as a commercially viable frontrunner for large-scale waste heat recovery.

Beyond thermoelectric metrics, mechanical robustness is a prerequisite for device fabrication and long-term reliability. The lattice repair strategy yields a significant “side benefit”: trace Ba doping increases the Vickers hardness from ∼550 MPa to ∼670 MPa (Figure S12). This ∼22% enhancement stems from three synergistic mechanisms: (1) Defect Healing: The repair of Mg vacancies restores the structural integrity of the cation sublattice; (2) Microstructure Strengthening: The formation of nanotwin boundaries creates a dense network that effectively impedes dislocation motion (Hall-Petch-like effect); and (3) Strain Hardening: The local compressive strain fields induced by Ba atoms enhance the interatomic bonding stiffness. This improved mechanical stability, coupled with excellent transport reproducibility (Figure S13), ensures high processability and durability during module assembly.

To validate the power generation potential, we fabricated a single-leg thermoelectric generator using the optimized material. Benefiting from its excellent chemical inertness and thermodynamic stability, niobium foil (∼30 μm) was selected as the hot-side barrier layer. This choice not only prevents the formation of resistive interfacial reaction layers but also effectively buffers thermal stress, ensuring a robust, low-resistance contact.^65^ EDS analysis (Figure S14) confirms a sharp interface with negligible diffusion, resulting in an ultralow contact resistivity of ∼11.5 μΩ·cm^2^ (Figure S15). We systematically evaluated the device performance with the cold side fixed at ∼300 K. The voltage–current (*U*–*I*) curves exhibit excellent linearity across all temperature gradients, indicating stable ohmic contact. As the hot-side temperature (*T*_h_) increases to 773 K, the device delivers a maximum output power (*P*_max_) of ∼0.23 W (Figure 6c). Most notably, the conversion efficiency (η), calculated using the measured heat flow (*Q*_c_), reaches a peak of ∼14% (Figure S16 and Figure 6d). This high efficiency, achieved in a single-leg configuration, serves as a definitive proof-of-concept for the practical deployment of lattice-repaired Mg_3_Sb_2_ in mid-to-high temperature power generation.

## 3 Conclusions

In summary, we have successfully demonstrated a “lattice repair” strategy rooted in thermodynamic defect engineering to resolve the intrinsic mobility-thermal conductivity trade-off in n-type Mg_3_(Sb,Bi)_2_. By targeting the labile cation sublattice with homologous Ba substitution, we achieved a dual-functional regulation of the crystal lattice. On one hand, the thermodynamic stabilization elevates the vacancy formation energy from ∼0.97 eV to ∼2.42 eV, effectively ″purifying″ the lattice from ionized impurity scattering centers and restoring carrier mobility by ∼35%. On the other hand, the heavy dopant introduces a hierarchical scattering architecture—ranging from atomic-scale mass fluctuations and local strain fields to mesoscale nanotwins—which creates a full-spectrum barrier for phonon propagation, driving the lattice thermal conductivity to an ultralow limit of ∼0.4 W m^–1^ K^–1^. This precise realization of the ″phonon-glass electron-crystal″ concept yields a record-breaking peak *zT* of 2.13 and a commercially viable average *zT*_ave_ of ∼1.5. The practical potential is further validated by a single-leg generator achieving a high conversion efficiency of ∼14%, underpinned by enhanced mechanical robustness and low material costs. Beyond the specific performance breakthroughs, this work establishes a generalizable paradigm for Zintl phase thermoelectrics: regulating intrinsic defect energetics via isovalent substitution offers a fundamental pathway to decouple electron and phonon transport, paving the way for scalable and cost-effective waste heat recovery technologies.

## Supplementary Material



## Data Availability

The data supporting the findings of this study are available in the Zenodo repository at https://doi.org/10.5281/zenodo.18831830. Additional information is provided in the Supporting Information.

## Notes

Experimental procedures for synthesis, characterization, and device assembly; additional structural analyses including XRD patterns and Rietveld refinements; temperature-dependent thermoelectric transport properties (conductivity, Seebeck coefficient, Hall data, and weighted mobility); detailed microstructural characterization via TEM and aberration-corrected STEM (ABF/HAADF imaging, GPA strain mapping, and elemental distributions); mechanical and thermal properties (Vickers hardness and heat capacity); positron annihilation lifetime spectroscopy (PALS) data; device performance metrics (contact resistivity, output characteristics, and reproducibility); and a comprehensive cost/performance comparison of n-type thermoelectric materials (PDF)
