# Supplementary material for: Defect-Energy-Targeted Lattice Repair Delivers High Thermoelectric Performance in Magnesium Antimonide
Source: J Am Chem Soc. 2026 Mar 14;148(11):12333–42. doi: 10.1021/jacs.6c02279 (PMC13022861; doi:10.1021/jacs.6c02279)
Supplement: Supplementary file 1 [file ja6c02279_si_001.pdf]

Supporting Information for

Defect-Energy-Targeted Lattice Repair Delivers High Thermoelectric Performance in Magnesium Antimonide†

Authors: Jiahao Jiang[1,5], Minhui Yuan[1,2,5], Yuntian Fu[3],Yanqi Huang[1], WenJie Li[1], Jingyi Lyu[1], Zeqing Hu[1], Shenghua Liu[1], Ran He[2*], Yanglong Hou[1,4*], Jing Shuai[1,4*]

Affiliations:

[1]*School of Materials, Shenzhen Campus of Sun Yat-sen University, Shenzhen 518107, China*

[2]*Leibniz Institute for Solid State and Materials Research IFW Dresden, Dresden 01069, Germany*

[3]*State Key Laboratory for Modification of Chemical Fibers and Polymer, Materials & College of Materials Science and Engineering, Donghua University, Shanghai 201620, China*

[4]*State Key Laboratory of Optoelectronic Materials and Technologies, Sun Yat-Sen University, Guangzhou 510275, China*

[5]*These authors contributed equally to this work*

[*]Correspondence: r.he@ifw-dresden.de; hou@mail.sysu.edu.cn; shuaij3@mail.sysu.edu.cn.

Materials and Methods

Materials Synthesis

High-purity raw materials including Mg turnings (99.9%; Aladdin), Ca powder (99.5%; Aladdin), Sr powder (99.9%; Macklin), Ba pieces (99.9%; Aladdin), Sb shots (99.999%; Aladdin), Bi shots (99.999%; Aladdin), and Te pieces (99.999%; Aladdin) were used as starting materials. Stoichiometric amounts of these elements were weighed according to the nominal compositions $Mg_{3.2}A_{0.005}Sb_{1.5}Bi_{0.49}Te_{0.01}$ (A=Ca, Sr, Ba) and $Mg_{3.2}Ba_xSb_{1.5}Bi_{0.49}Te_{0.01}$ (x = 0, 0.001, 0.003, 0.005, 0.007) in a glove box under Ar atmosphere with oxygen and water content below 0.1ppm. The weighed materials were loaded into a stainless steel ball mill jar and carried out ball milling for 5 hours in a high-energy ball mill(SPEX Sample Prep, 8000M, America). The resulting powder was subsequently loaded into a graphite die and consolidated using spark plasma sintering (SPS, FUJI-212Lx, Japan) at 1073 K under 50 MPa pressure for 2 min in an argon atmosphere.

Phase and Microstructure Characterizations

The phase structure of all samples was characterized by X-ray diffraction (XRD, Bruker D8, Germany) using Cu Kα radiation (λ = 1.5403 Å) operating at 40 kV and 40 mA. Data were collected with a step size of 0.02°. The bulk XRD patterns were subjected to Rietveld refinement using the General Structure Analysis System (GSAS) to analyze the crystal structure. Electron backscattered diffraction (EBSD) measurements were performed using a scanning electron microscope (SEM, Thermo Scientific Apreo 2C, Czech Republic) operated at 20 kV with a step size of 0.2 μm. Samples for EBSD observation were prepared by mechanical polishing to 0.1 μm

using diamond paste, followed by argon ion polishing using an ion beam milling system (IM4000II, Japan). Grain size statistics were derived from the analysis of approximately 100 grains per sample using the AZtecCrystal software. The surface morphology of polished samples was observed by SEM (Hitachi S-4800, Japan) operated at 30 kV equipped with an Energy Dispersive X-ray Spectroscopy (EDS) detector. Samples for transmission electron microscopy (TEM, FEI Tecnai F20, USA) and spherical aberration-corrected transmission electron microscopy (AC-TEM, Titan Cubed Themis G2300, USA) were prepared using a focused ion beam system (FIB, ZEISS Crossbeam 540, Germany). TEM and AC-TEM were operated at 120 kV and 200 kV, respectively, for microstructure characterization and EDS analysis. Fast Fourier transform (FFT), inverse fast Fourier transform (IFFT), and geometric phase analysis (GPA) images were obtained by processing the TEM images using Gatan Digital Micrograph (GMS-3) software. Vickers hardness was measured using a digital Vickers hardness tester (HV-1000, China), and each sample was tested three times.

Material Property Characterizations

The prepared samples were cut into rectangular columns with dimensions of approximately 2.5 × 2.5 × 10 mm^3 from the pressed discs. The electrical conductivity (σ) and Seebeck coefficient (S) were measured simultaneously using a ZEM-3 system (ULVAC-RIKO, Japan). The total thermal conductivity (κ_{tot}) was calculated using the equation $\kappa_{tot} = DC$□ρ, where D, C□, and ρ represent thermal diffusivity, specific heat capacity, and density, respectively. Thermal diffusivity (D) measurements were conducted on disc-shaped samples with dimensions of φ~10 mm × ~2 mm

using a laser flash analyzer (LFA-467 HT, Netzsch, Germany). The specific heat capacity (C□) was determined using the Dulong-Petit law and measured by differential scanning calorimetry (DSC 214 Polyma, Netzsch, Germany) at a heating rate of 5 K/min under N_2 atmosphere. Sample density (ρ) was measured using the Archimedes method with distilled anhydrous ethanol as the immersion medium, with measurements repeated five times to ensure accuracy. Hall coefficient measurements were performed as a function of temperature (300-773 K) using the van der Pauw technique under an applied magnetic field of 1.5 T. From these measurements, the carrier concentration (n_H) and Hall mobility (μ_H) were determined using the relations $n_H = 1/(eR_H)$ and $\mu_H = \sigma R_H$, respectively, where e is the elementary charge and R_H is the Hall coefficient. Sound velocity measurements were conducted using an Olympus EPOCH 650 ultrasonic system at ambient temperature. The average sound velocity (v_a) was calculated using the equation:

$$v_a^{-3} = 1/3\ (v_l^{-3} + 2v_t^{-3}) \tag{1}$$

where v_l is longitudinal sound velocity, and v_t is transverse sound velocity. The weighted mobility (μ_w) was calculated using the formula:

$$\mu_W = \frac{3h^3\sigma}{8\pi(2m_e k_B T)^{3/2}}\left[\frac{\exp[\frac{|S|}{k_B/e}-2]}{1+\exp[-5(\frac{|S|}{k_B/e}-1)]} + \frac{\frac{3}{\pi^2}\frac{|S|}{k_B/e}}{1+\exp[5\frac{|S|}{k_B/e}-1]}\right] \tag{2}$$

where k_B is the Boltzmann constant, h is the Planck constant, m_e is the electron mass, and e is the electron charge.

Module Fabrication and Efficiency Evaluation

For thermoelectric module fabrication, a multilayer structure was assembled in the following

sequence: Ni foil (0.1 mm thickness, 99.99% purity), Nb foil (0.03 mm thickness, 99.99% purity), $Mg_{3.2}Ba_{0.005}Sb_{1.5}Bi_{0.49}Te_{0.01}$ powder, followed by another layer of Nb foil and Ni foil. This assembly was loaded into a graphite mold for consolidation. The Nb foil served as a diffusion barrier to prevent interdiffusion of elements between the thermoelectric material and the metallic contacts. The Ni foil functioned as a metallization layer, providing electrical contact and ensuring interfacial stability between the thermoelectric material and external electrodes. The densification process was conducted using identical parameters to those employed for the sintering of $Mg_{3.2}Ba_{0.005}Sb_{1.5}Bi_{0.49}Te_{0.01}$ powder. After densification, the consolidated module was subjected to grinding and polishing procedures, followed by wire electrical discharge machining (wire-EDM) to obtain rectangular specimens with dimensions of 5 × 5 × 10 mm^3. For thermal and electrical characterization, both the hot and cold sides of the thermoelectric joints were soldered onto a double-sided direct bonded copper (DBC) alumina substrate using high-temperature silver paste (CT2700R7S). Copper wires were attached to the cold-side copper electrodes to enable current and voltage measurements during device operation. The energy conversion efficiency of the thermoelectric device was evaluated using the following relationship:

$$\eta = P / (P + Q_c) \times 100\% \quad (3)$$

where P represents the electrical output power of the thermoelectric device and Q_c denotes the heat flow rate measured using a heat flux sensor positioned at the cold end of the device.

DFT Calculation

All electronic band structure calculations were performed within the framework of density functional theory (DFT) as implemented in the Vienna Ab initio Simulation Package (VASP) code[1-3]. The exchange-correlation functional was treated using the Perdew-Burke-Ernzerhof (PBE) generalized gradient approximation[4] with the projector-augmented wave (PAW) method for describing the electron-ion interactions. The doped Mg_3Sb_2 systems were modeled using 3 × 3 × 2 supercells, and all atomic structures were fully relaxed until the forces on each atom were less than 0.01 eV/Å. A plane-wave cutoff energy of 400 eV was employed, and the Brillouin zone integration was performed using a Γ-centered 3 × 3 × 2 Monkhorst-Pack k-point mesh. To better visualize the band convergence effects in the doped systems, the electronic bands of the supercells were unfolded back into the first Brillouin zone of the primitive unit cell using the methodology implemented in the VASPKIT code[5]. The bonding characteristics were analyzed using the crystal orbital Hamilton population (COHP) method, which provides energy-resolved bonding and antibonding contributions for selected atomic pairs. These calculations were performed using the LOBSTER software package[6-9]. Phonon dispersion relations and phonon group velocities were calculated using the finite-difference supercell approach as implemented in the PHONOPY code[10-12]. The phonon calculations were based on 4 × 4 × 2 supercells with force calculations performed using a 3 × 3 × 3 Monkhorst-Pack k-point mesh.

Point defect formation energies were calculated using the standard supercell approach with 3 × 3 × 2 supercells containing 90 atoms. The formation energy of a Mg vacancy in pristine Mg_3Sb_2 was

calculated according to:

$$E_f = E(Mg_{53}Sb_{36}) - E(Mg_{54}Sb_{36}) + \mu(Mg)$$

where $E(Mg_{53}Sb_{36})$ and $E(Mg_{54}Sb_{36})$ represent the total energies of a defective and a perfect supercell, respectively, and μ denotes the chemical potential of an element in its bulk phase.

For the Ba(Sr,Ca)-doped Mg_3Sb_2 alloys, the formation energy of a Mg vacancy in the presence of alkaline earth dopants was calculated using:

$$E_{V_{Mg}-Ba_{Mg}} = E(BaMg_{52}Sb_{36}) - E(BaMg_{53}Sb_{36}) + \mu(Mg)$$

The calculations reveal that alloying Mg_3Sb_2 with Ba, Sr, or Ca significantly increases the Mg vacancy formation energy, indicating that Mg vacancy formation becomes energetically less favorable upon alkaline earth element incorporation. This effect is most pronounced for Ba doping, which is consistent with the experimentally observed reduction in hole carrier concentration upon Ba addition to the Mg_3Sb_2 matrix.

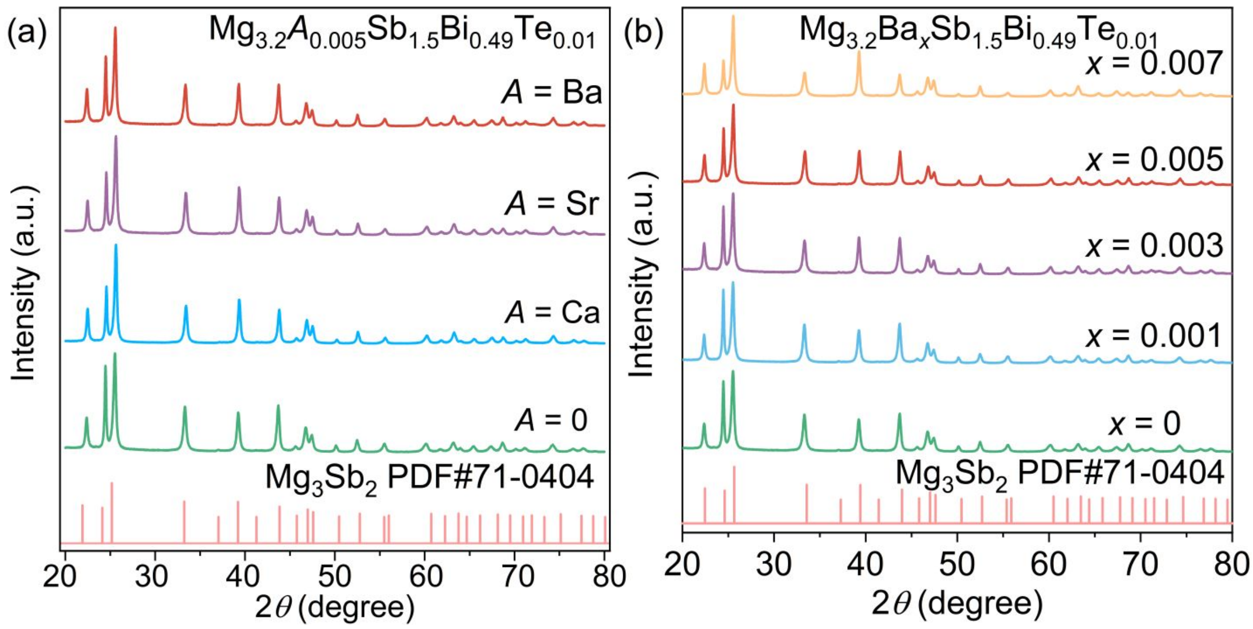

Figure S1. Room-temperature X-ray diffraction (XRD) patterns of (a) $Mg_{3.2}A_{0.005}Sb_{1.5}Bi_{0.49}Te_{0.01}$ (A = Ca, Sr, Ba) and (b) $Mg_{3.2}Ba_xSb_{1.5}Bi_{0.49}Te_{0.01}$ (x = 0, 0.001, 0.003, 0.005, 0.007) samples.

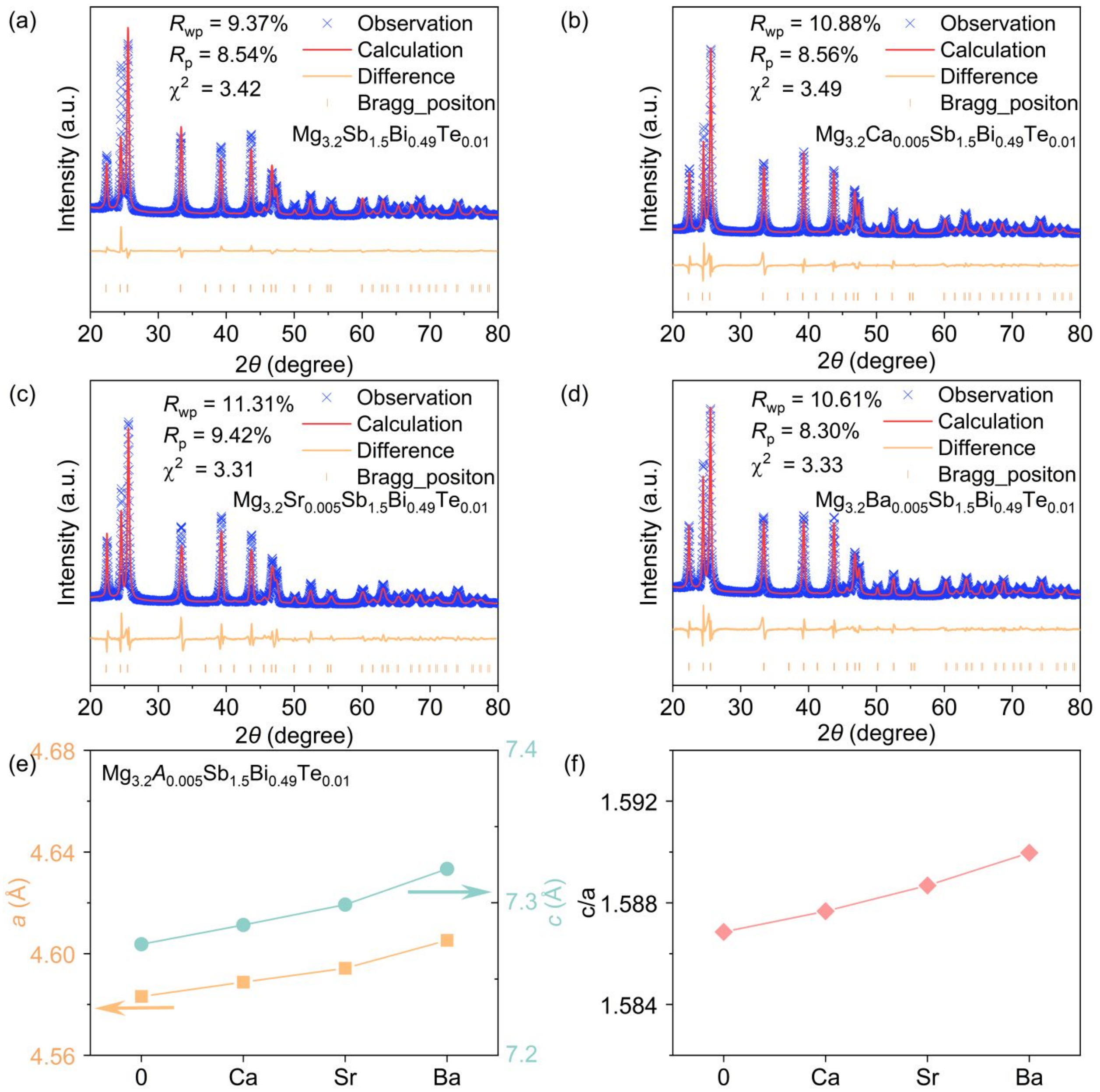

Figure S2. Rietveld refinement results for $Mg_{3.2}A_{0.005}Sb_{1.5}Bi_{0.49}Te_{0.01}$ samples. (a-d) XRD refinement profiles for A = 0, Ca, Sr, and Ba, respectively. (e) Calculated lattice parameters and (f) *c*/*a* ratio as a function of dopant type.

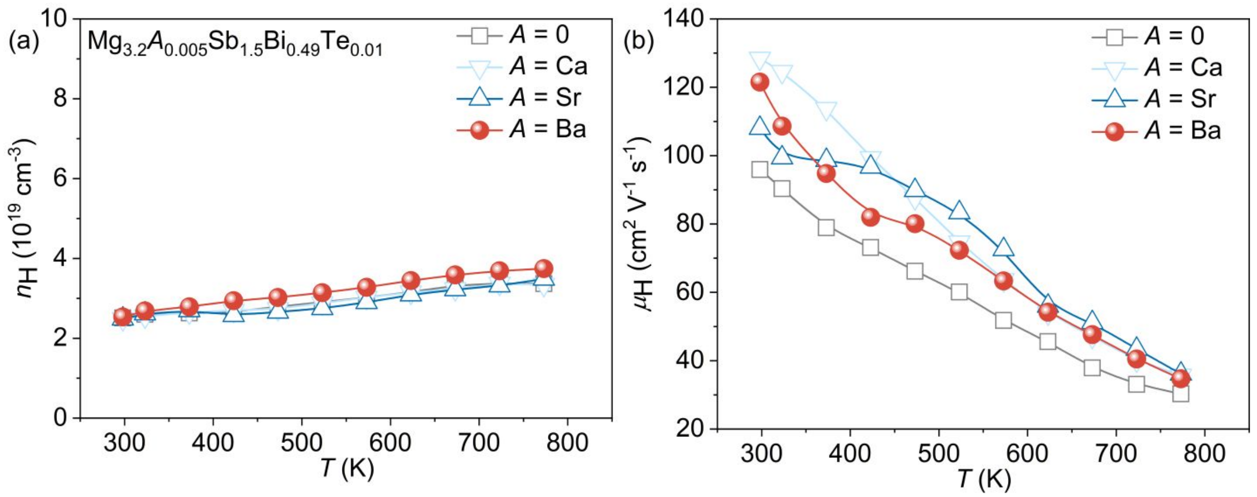

Figure S3. Temperature-dependent (a) carrier concentration and (b) carrier mobility of $Mg_{3.2}A_{0.005}Sb_{1.5}Bi_{0.49}Te_{0.01}$ (A = 0, Ca, Sr, Ba) samples.

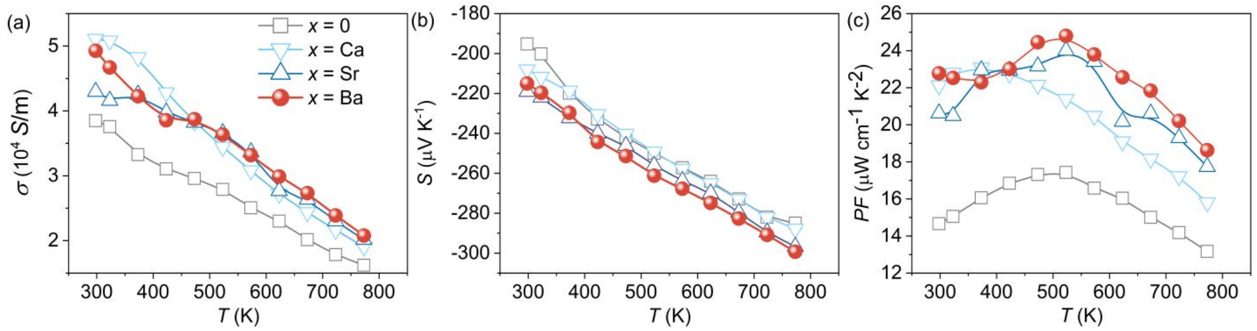

Figure S4. The temperature-dependent thermoelectric properties of $Mg_{3.2}A_{0.005}Sb_{1.5}Bi_{0.49}Te_{0.01}$ (A = 0, Ca, Sr, Ba) samples: (a) electrical conductivity, (b) Seebeck coefficient, and (c) power factors.

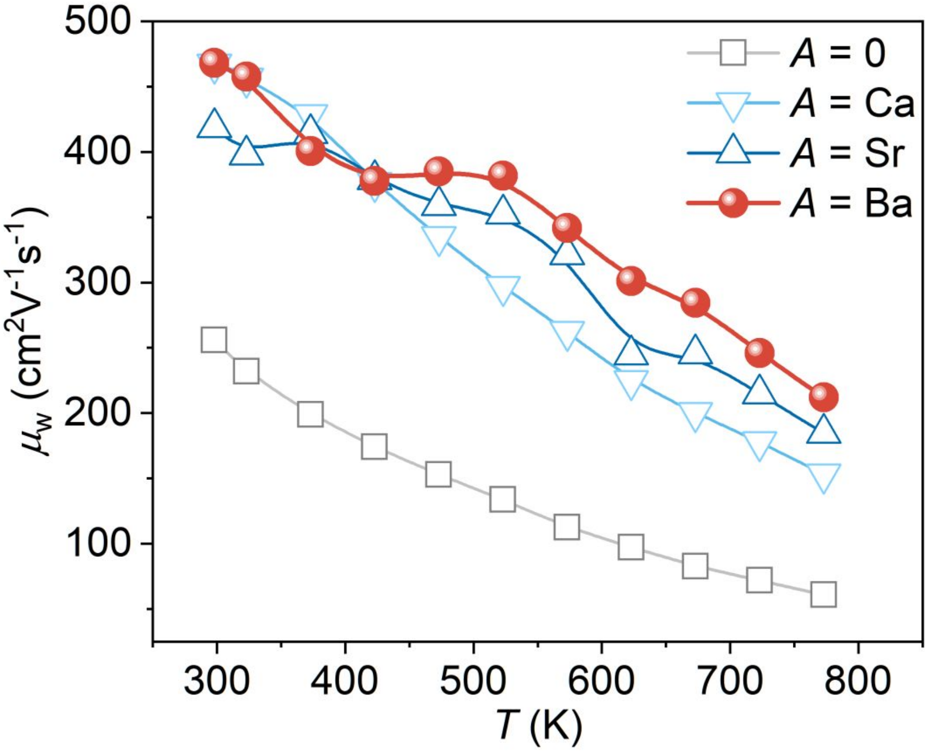

Figure S5. The temperature-dependent weighted mobility of $Mg_{3.2}A_{0.005}Sb_{1.5}Bi_{0.49}Te_{0.01}$ (A = 0, Ca, Sr, Ba) samples.

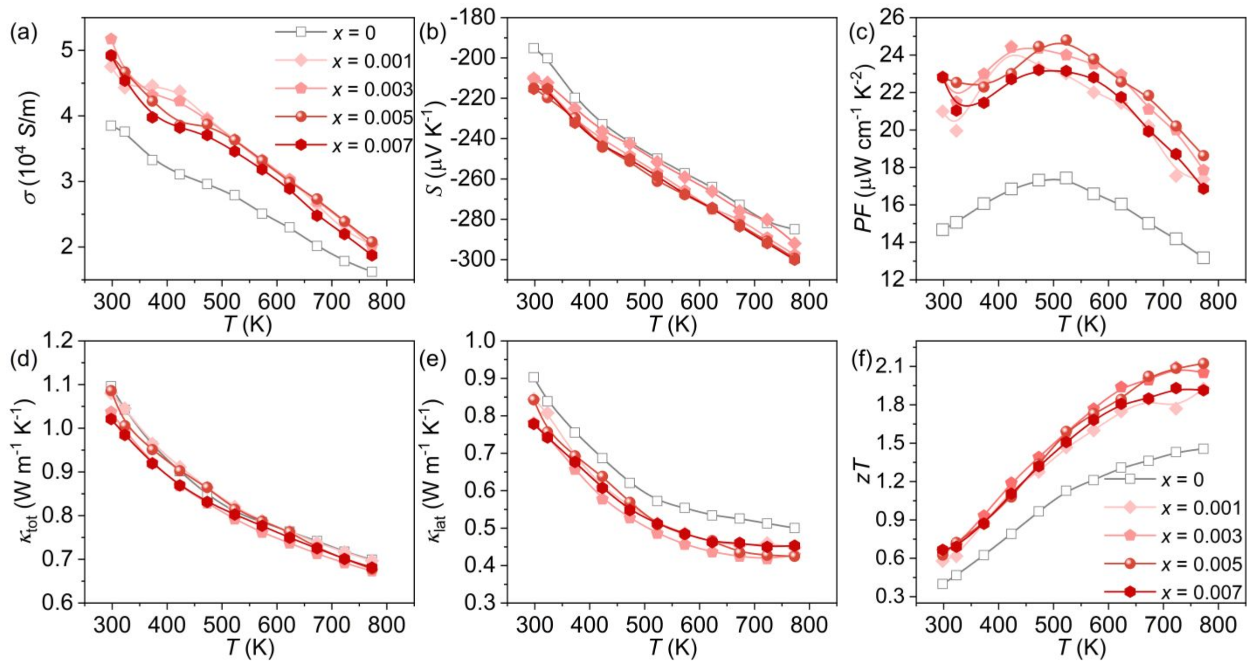

Figure S6. Thermoelectric transport properties of $Mg_{3.2}Ba_xSb_{1.5}Bi_{0.49}Te_{0.01}$ (x = 0, 0.001, 0.003, 0.005, 0.007) samples as a function of temperature: (a) electrical conductivity (σ), (b) Seebeck coefficient (S), (c) power factor (PF), (d) total thermal conductivity (κ_{tot}), (e) lattice thermal conductivity (κ_{L}), and (f) dimensionless figure of merit (ZT).

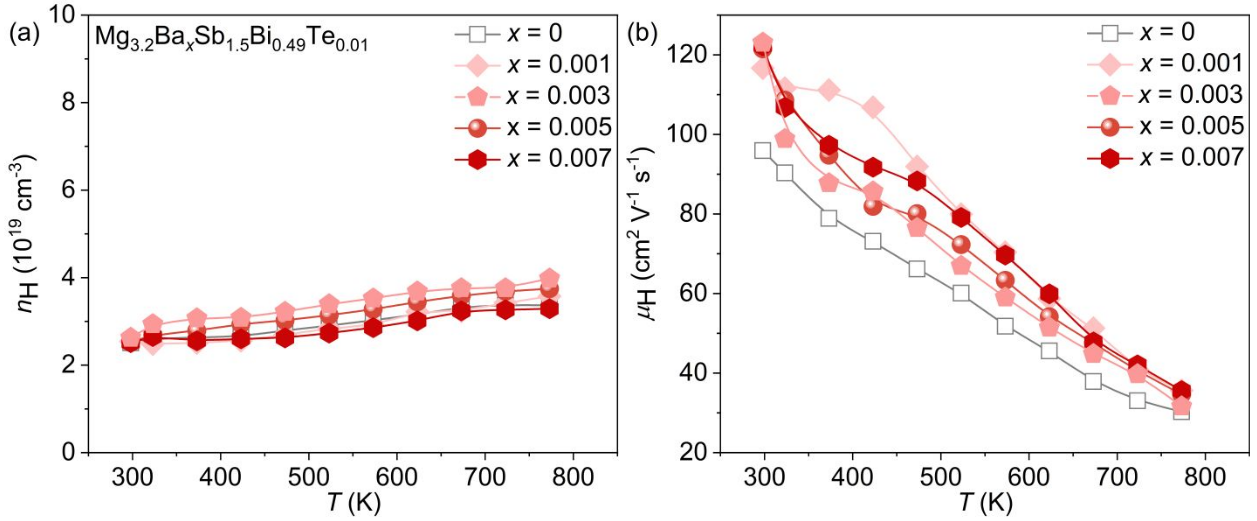

Figure S7. The temperature-dependent (a) carrier concentration and (b) carrier mobility of $Mg_{3.2}Ba_xSb_{1.5}Bi_{0.49}Te_{0.01}$ (x = 0, 0.001, 0.003, 0.005, 0.007) samples.

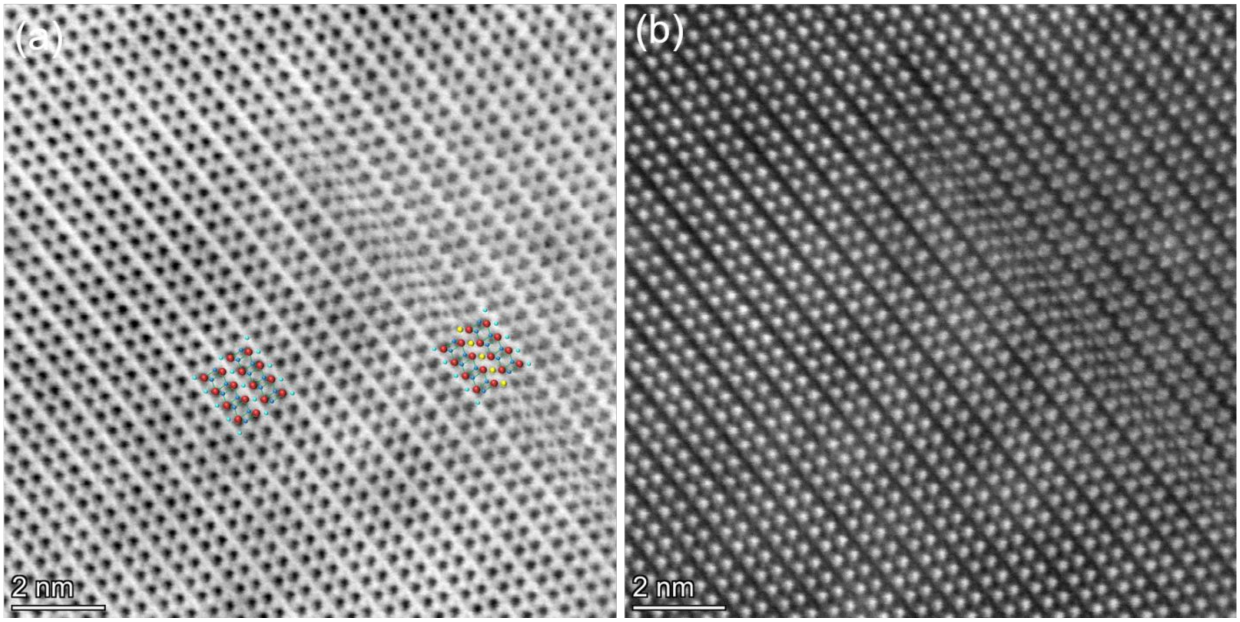

Figure S8. High-resolution scanning transmission electron microscopy (STEM) analysis of the $Mg_{3.2}Ba_{0.005}Sb_{1.5}Bi_{0.49}Te_{0.01}$ sample. (a) Annular bright-field (ABF)-STEM image. (b) High-angle annular dark-field (HAADF)-STEM image used for geometric phase analysis (GPA) presented in Figure 5(d).

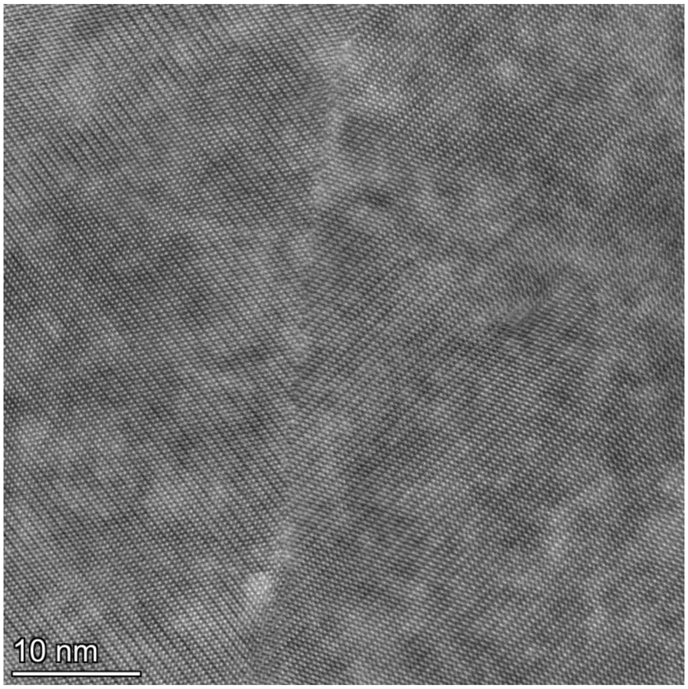

Figure S9. High-resolution STEM image showing twin crystal structures in the $Mg_{3.2}Ba_{0.005}Sb_{1.5}Bi_{0.49}Te_{0.01}$ sample.

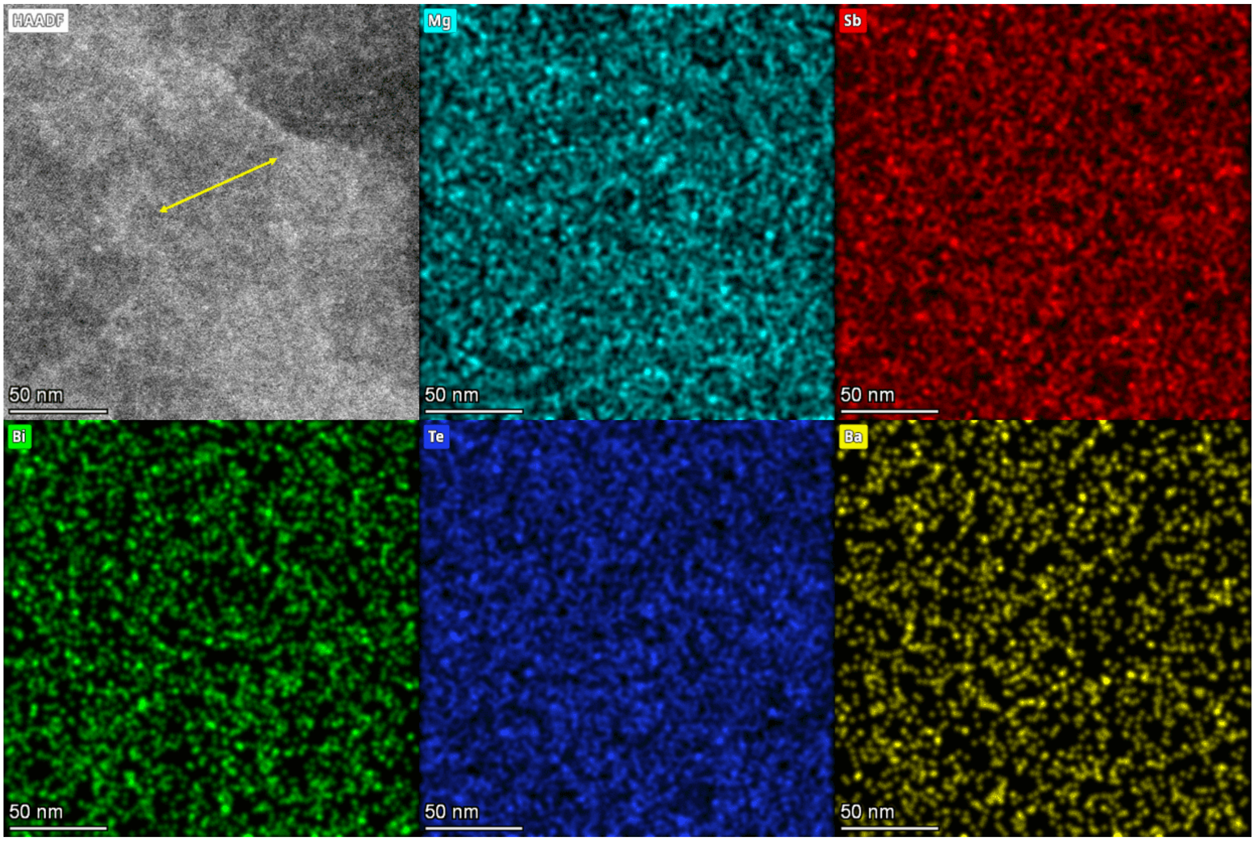

Figure S10. EDS-TEM elemental mapping of the $Mg_{3.2}Ba_{0.005}Sb_{1.5}Bi_{0.49}Te_{0.01}$ sample showing the distribution of Mg, Ba, Sb, Bi, and Te elements.

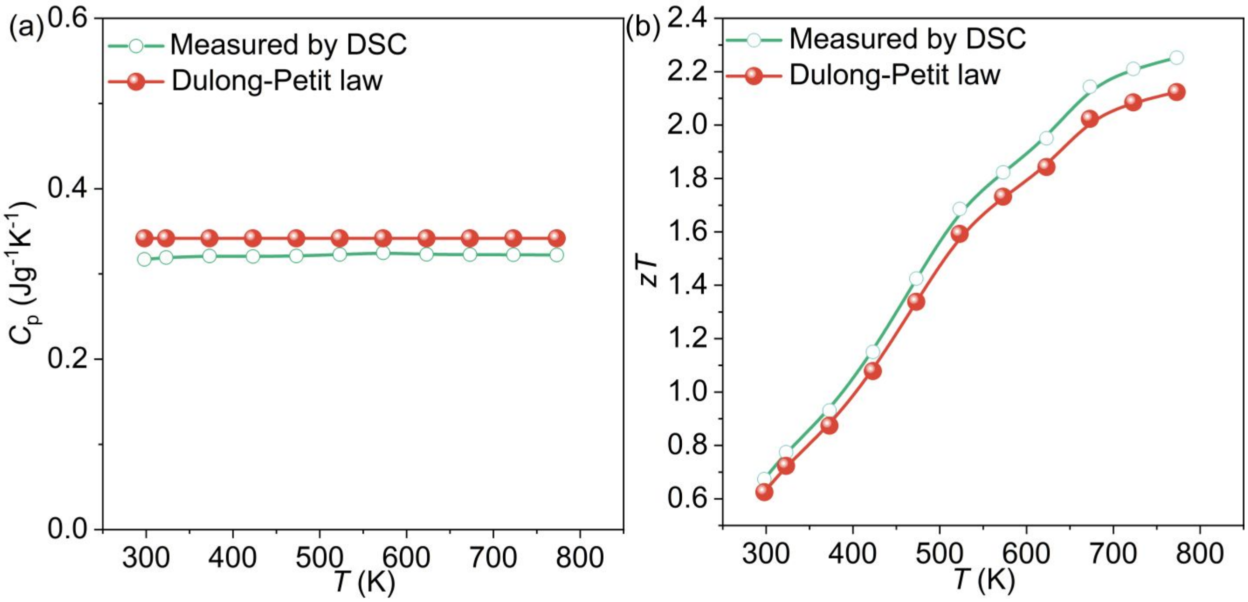

Figure S11. (a) Comparison of temperature-dependent heat capacity (C_p) for $Mg_{3.2}Ba_{0.005}Sb_{1.5}Bi_{0.49}Te_{0.01}$ sample, determined by the Dulong-Petit law and differential scanning calorimetry (DSC) measurements. (b) Temperature-dependent zT values calculated using different C_p data sources. A maximum zT of ~2.25 at 773 K was achieved when the DSC-derived C_p values were used for thermal conductivity calculations.

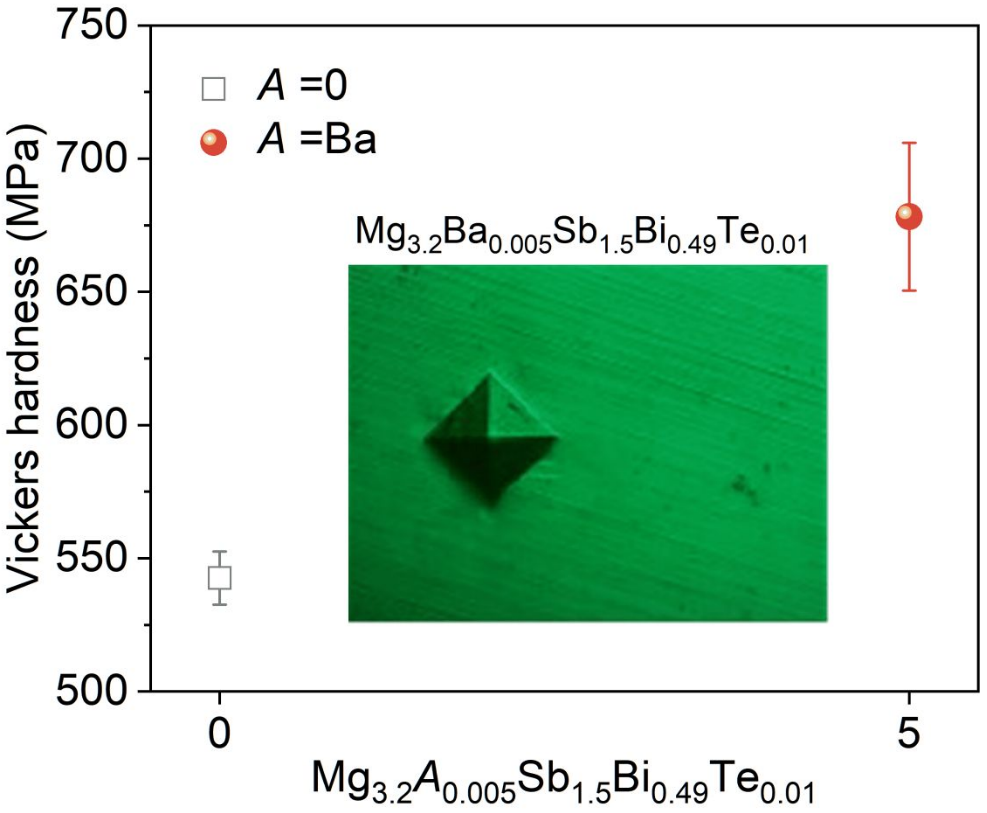

Figure S12. Vickers hardness comparison between pristine $Mg_{3.2}A_{0.005}Sb_{1.5}Bi_{0.49}Te_{0.01}$ (A = 0) and Ba-doped $Mg_{3.2}Ba_{0.005}Sb_{1.5}Bi_{0.49}Te_{0.01}$ samples.

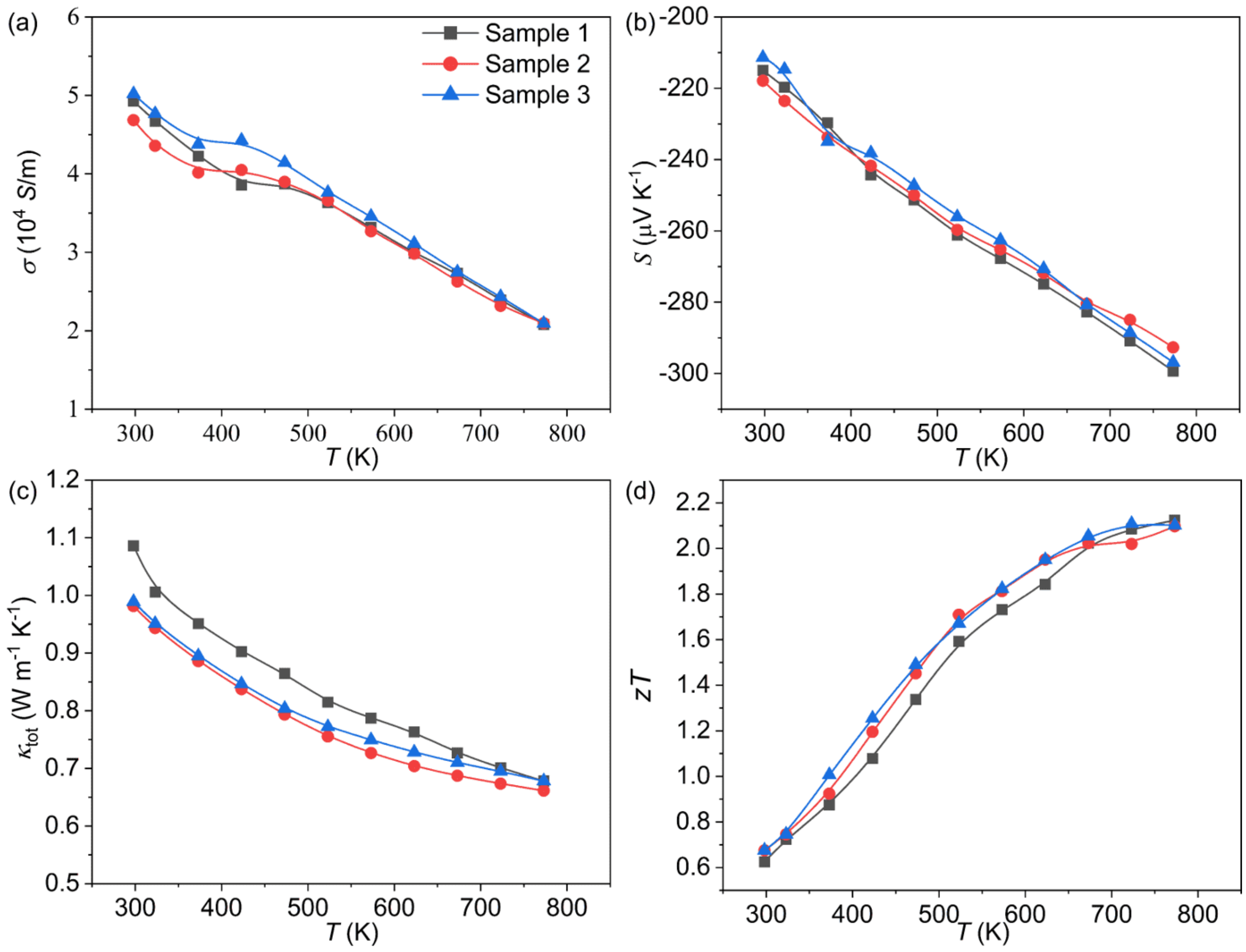

Figure S13. Reproducibility assessment of thermoelectric transport properties for $Mg_{3.2}Ba_{0.005}Sb_{1.5}Bi_{0.49}Te_{0.01}$ samples measured three times: (a) electrical conductivity (σ), (b) Seebeck coefficient (S), (c) total thermal conductivity (κ_{tot}), and (d) dimensionless figure of merit (ZT).

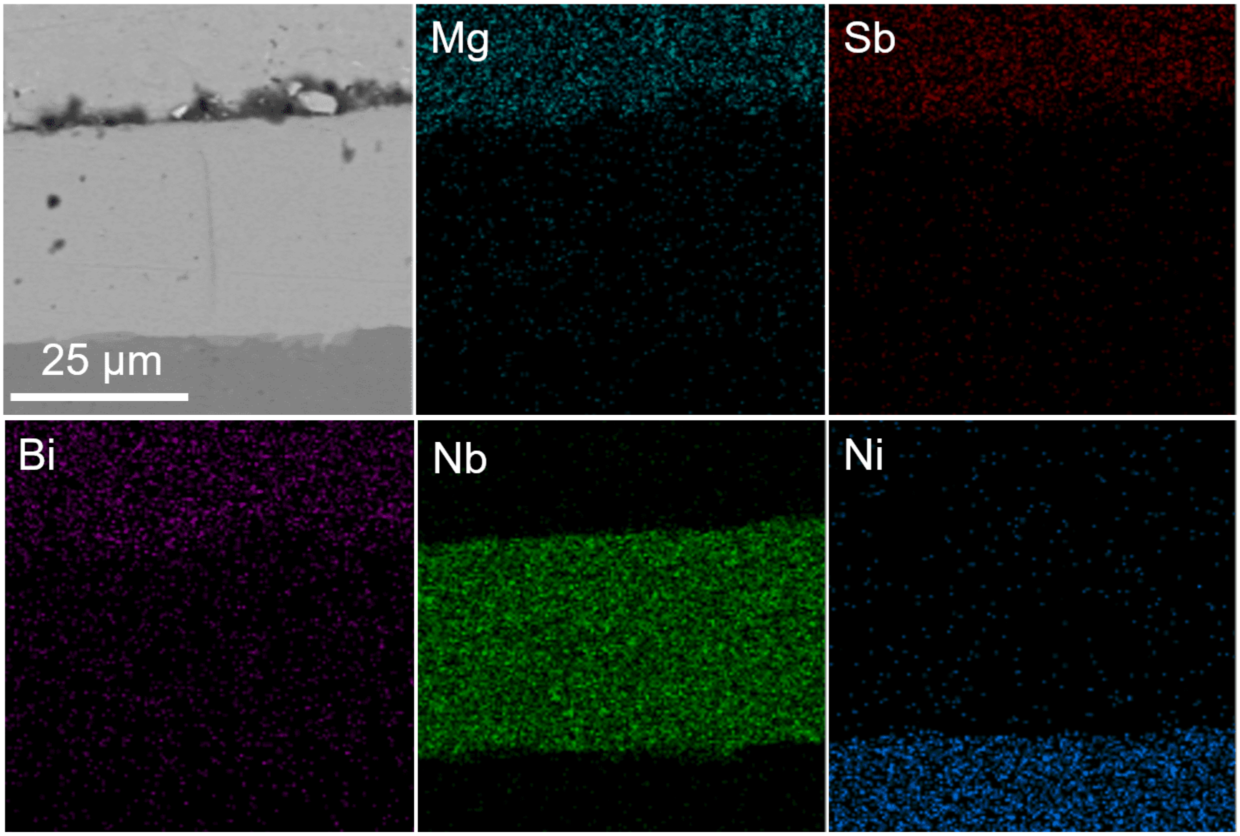

Figure S14. Surface morphology and elemental distribution of the polished $Mg_{3.2}Ba_{0.005}Sb_{1.5}Bi_{0.49}Te_{0.01}$ thermoelectric leg, showing the corresponding EDS mapping results.

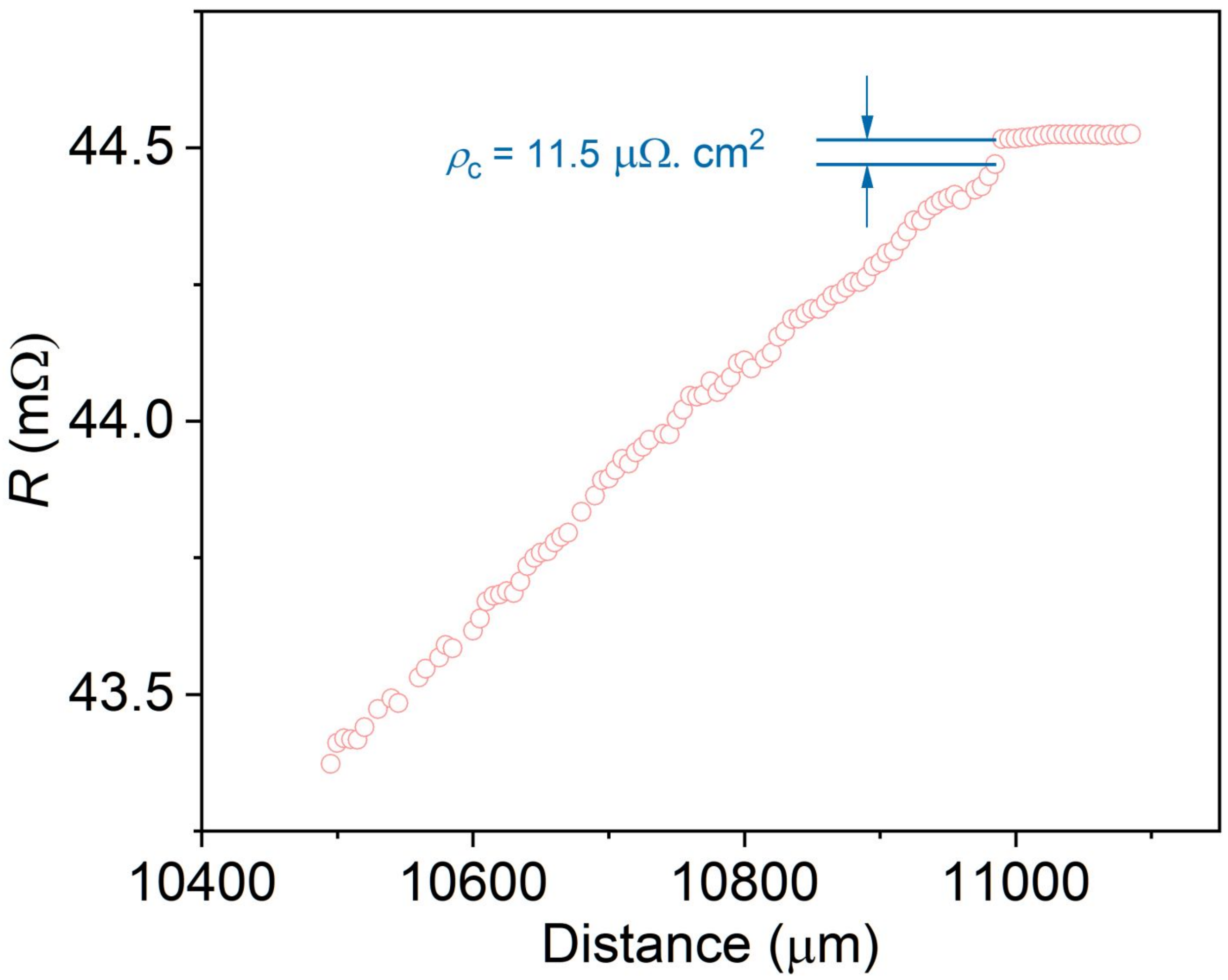

Figure S15. Room-temperature contact resistivity measurements of the $Mg_{3.2}Ba_{0.005}Sb_{1.5}Bi_{0.49}Te_{0.01}$ thermoelectric leg.

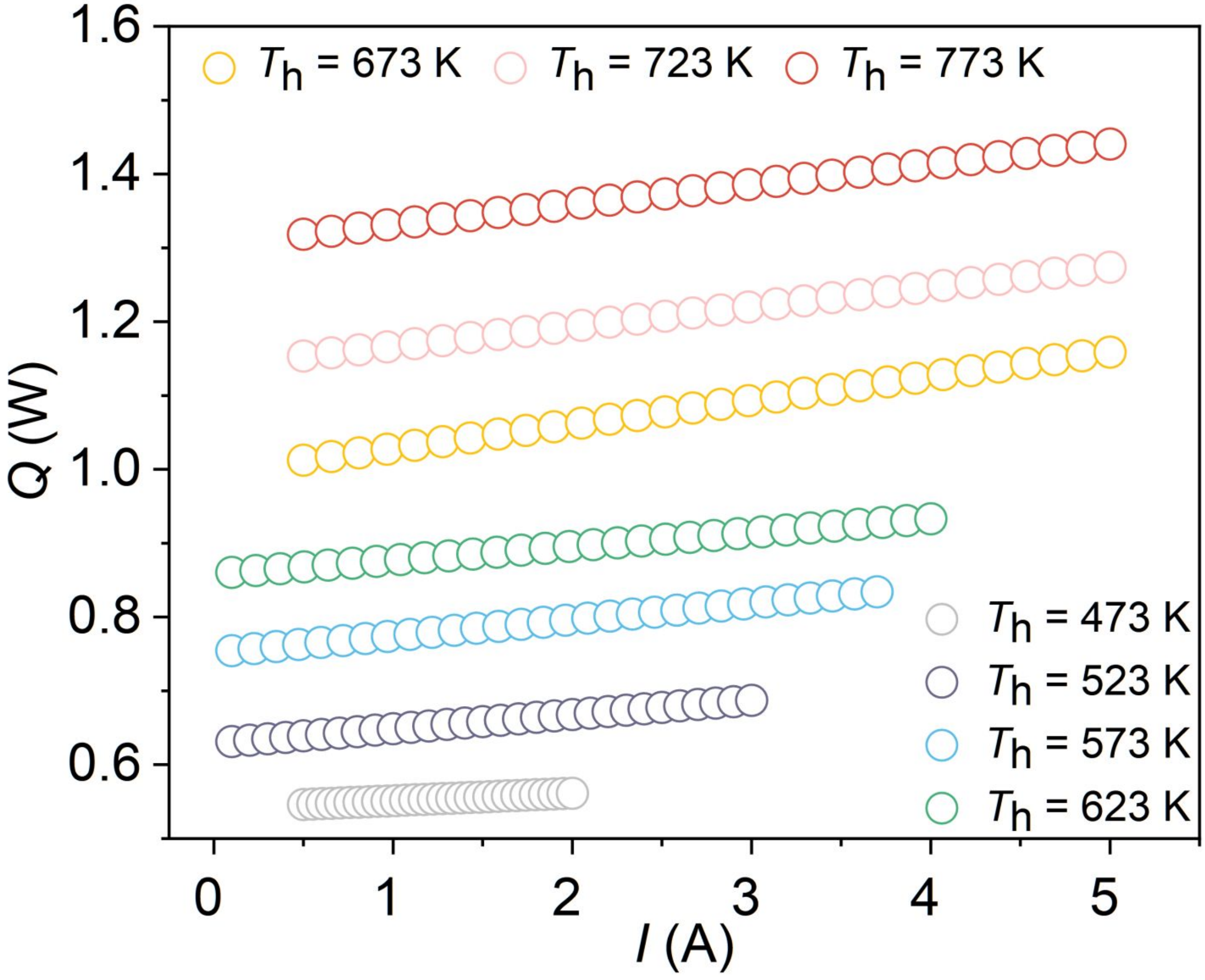

Figure S16. Relationships between heat flow and applied current for the $Mg_{3.2}Ba_{0.005}Sb_{1.5}Bi_{0.49}Te_{0.01}$ single-leg thermoelectric device at various hot-side temperatures.

Supplementary Table S1. Comparison of positron lifetimes and relative intensities for $Mg_{3.2}A_{0.005}Sb_{1.5}Bi_{0.49}Te_{0.01}$ (A = 0, Ba) samples obtained from positron annihilation lifetime spectroscopy (PALS) measurements.

Sample	τ_1 (ps)	τ_2 (ps)	I_1	I_2	χ^2
A = 0	135.1	337.6	85.6	14.4	1.07
A = Ba	143.5	358.1	91.4	8.6	1.06

Supplementary Table S2. Refined structural parameters of the pristine $Mg_{3.2}Sb_{1.5}Bi_{0.49}Te_{0.01}$ sample obtained from Rietveld refinement analysis. During refinement, the occupancy of the Mg(2) site was fixed at full occupancy (1.0), while Sb, Bi, and Te atoms were constrained to share the same crystallographic position with identical isotropic displacement parameters (U_{ios}).

Atom	Site	Occupancy	x	y	z	U_{ios} (Å^2)
Mg(1)	1*a*	0.9153(9)	0	0	0	0.051
Mg(2)	2*d*	1	1/3	2/3	0.6403	0.002
Sb	2*d*	0.75	1/3	2/3	0.2302	0.0048
Bi	2*d*	0.245	1/3	2/3	0.2302	0.0048
Te	2*d*	0.005	1/3	2/3	0.2302	0.0048

Supplementary Table S3. Refined structural parameters of the Ba-doped $Mg_{3.2}Ba_{0.005}Sb_{1.5}Bi_{0.49}Te_{0.01}$ sample obtained from Rietveld refinement analysis.

Atom	Site	Occupancy	*x*	*y*	*z*	U_{ios} (Å^2)
Mg(1)	1*a*	0.9341(1)	0	0	0	0.05929
Mg(2)	2*d*	1	1/3	2/3	0.6375	0.018
Sb	2*d*	0.75	1/3	2/3	0.2276	0.0107
Bi	2*d*	0.245	1/3	2/3	0.2276	0.0107
Te	2*d*	0.005	1/3	2/3	0.2276	0.0107

Supplementary Table S4. Carrier concentration, carrier mobility (determined by Hall measurements), and density of $Mg_{3.2}A_xSb_{1.5}Bi_{0.49}Te_{0.01}$ samples.

Samples	Carrier concentration (cm^{-3})	Carrier mobility ($cm^2V^{-1}s^{-1}$)	Density (g/cm^3)
A = 0; x = 0	2.51×10^{19}	95.92	4.416
A = Ca; x = 0.005	2.49×10^{19}	116.68	4.452
A = Sr; x = 0.005	2.49×10^{19}	116.43	4.450
A = Ba; x = 0.005	2.54×10^{19}	121.50	4.437
A = Ba; x = 0.001	2.55×10^{19}	116.66	4.444
A = Ba; x = 0.003	2.58×10^{19}	123.07	4.452
A = Ba; x = 0.007	2.52×10^{19}	122.27	4.441

Supplementary Table S5. Cost analysis of the constituent elements employed in this study. The purity of all elements is 99.99% or higher. Data acquisition date: October 5, 2025. Data sources: https://www.chemicalbook.com, and https://www.shmet.com and https://china.guidechem.com.

Element	Price (USD/kg)	Element	Price (USD/kg)
Mg	2.46	Ag	1155.91
Sb	31.18	Ge	4.53
Bi	20.26	Cu	12.02
Te	100.96	Co	35.71
Sn	41.69	Si	2.46
Se	39.84	Bi_2O_3	9.34
Pb	2.34	S	3.43

Supplementary Table S6. Cost comparison of n-type thermoelectric materials investigated in this work, including operating temperature ranges and average dimensionless figure of merit (zT_{ave}) values.

Materials	Price (USD/kg)	Test temperature ranges (K)	zT_{ave}	Ref.
$Mg_3Sb_{1.5}Bi_{0.5}$	22.20	300-773	1.47	This work
$Bi_2Te_{2.7}Se_{0.3}$	56.20	300-523	1.38	[13]
$Mg_3Bi_{1.5}Sb_{0.5}$	18.84	300-600	1.3	[14]
$CoSb_3$	31.18	300-850	1.12	[15]
PbTe	39.91	323-773	1.04	[16]
SiGe	3.68	300-1073	1.02	[17]
$Mg_2Si_{0.3}Sn_{0.7}$	25.67	300-800	1	[18]
PbSe	12.69	300-823	1	[19]
BiCuSeO	18.43	373-773	0.84	[20]
PbS	2.49	300-900	0.8	[21]
SnSe	40.95	300-873	0.76	[22]
SnTe	72.40	300-823	0.51	[23]
GeTe	65.97	300-673	0.36	[24]
SnS	33.56	323-823	0.16	[25]

Reference

(1) Kresse, G.; Furthmuller, J. Efficiency of ab-initio total energy calculations for metals and semiconductors using a plane-wave basis set. *Comput. Mater. Sci.* **1996**, *6* (1), 15-50.

(2) Kresse; Furthmuller. Efficient iterative schemes for ab initio total-energy calculations using a plane-wave basis set. *Phys. Rev. B: Condens. Matter* **1996**, *54* (16), 11169-11186.

(3) Blochl. Projector augmented-wave method. *Phys. Rev. B: Condens. Matter* **1994**, *50* (24), 17953-17979.

(4) Perdew; Burke; Ernzerhof. Generalized Gradient Approximation Made Simple. *Phys. Rev. Lett.* **1996**, *77* (18), 3865-3868.

(5) Wang, V.; Xu, N.; Liu, J.-C.; Tang, G.; Geng, W.-T. VASPKIT: A user-friendly interface facilitating high-throughput computing and analysis using VASP code. *Comput. Phys. Commun.* **2021**, *267*, 1108033.

(6) Dronskowski, R.; Blochl, P. E. Crystal orbital Hamilton populations (COHP). Energy-resolved visualization of chemical bonding in solids based on density-functional calculations. *J. Phys. Chem.* **1993**, *97* (33), 8617-8624.

(7) Deringer, V. L.; Tchougreeff, A. L.; Dronskowski, R. Crystal Orbital Hamilton Population (COHP) Analysis As Projected from Plane-Wave Basis Sets. *J. Phys. Chem. A* **2011**, *115* (21), 5461-5466.

(8) Maintz, S.; Deringer, V. L.; Tchougreeff, A. L.; Dronskowski, R. Analytic projection from plane-wave and PAW wavefunctions and application to chemical-bonding analysis in solids. *J. Comput. Chem.* **2013**, *34* (29), 2557-2567.

(9) Nelson, R.; Ertural, C.; George, J.; Deringer, V. L.; Hautier, G.; Dronskowski, R. LOBSTER: Local orbital projections, atomic charges, and chemical-bonding analysis fromprojector-augmented-wave-baseddensity-functional theory. *J. Comput. Chem.* **2020**, *41* (21), 1931-1940.

(10) Wu, X.; Vanderbilt, D.; Hamann, D. R. Systematic treatment of displacements, strains, and electric fields in density-functional perturbation theory. *Phys. Rev. B* **2005**, *72* (3), 035105.

(11) Huang, L.-F.; Zeng, Z. Roles of Mass, Structure, and bond strength in the phonon properties

and lattice anharmonicity of single-layer Mo and W dichalcogenides. *J. Phys. Chem. C* **2015**, *119* (32), 18779-18789.

(12) Togo, A.; Tanaka, I. First principles phonon calculations in materials science. *Scr. Mater.* **2015**, *108*, 1-5.

(13) Zhu, B.; Liu, X. X.; Wang, Q.; Qiu, Y.; Shu, Z.; Guo, Z. T.; Tong, Y.; Cui, J.; Gu, M.; He, J. Q. Realizing record high performance in n-type Bi_2Te_3-based thermoelectric materials. *Energy Environ. Sci.* **2020**, *13* (7), 2106-2114.

(14) Song, W.; Sun, C.; Yang, K.; Ke, S.; Li, X.; Liu, M.; Sun, C.; Zhao, W.; Zhang, Q. Magnetism-induced diffuse scattering effect triggers excellent thermoelectric performance. *Energy Environ. Sci.* **2024**, *17* (14), 5216-522.

(15) Zhao, W. Y.; Liu, Z. Y.; Sun, Z. G.; Zhang, Q. J.; Wei, P.; Mu, X.; Zhou, H. Y.; Li, C. C.; Ma, S. F.; He, D. Q.; et al. Superparamagnetic enhancement of thermoelectric performance. *Nature* **2017**, *549* (7671), 247-251.

(16) Jia, B. H.; Huang, Y.; Wang, Y.; Zhou, Y. S. Y.; Zhao, X. D.; Ning, S. T.; Xu, X.; Lin, P. J.; Chen, Z. Q.; Jiang, B. B.; et al. Realizing high thermoelectric performance in non-nanostructured n-type PbTe. *Energy Environ. Sci.* **2022**, *15* (5), 1920-1929.

(17) Basu, R.; Bhattacharya, S.; Bhatt, R.; Roy, M.; Ahmad, S.; Singh, A.; Navaneethan, M.; Hayakawa, Y.; Aswal, D. K.; Gupta, S. K. Improved thermoelectric performance of hot pressed nanostructured n-type SiGe bulk alloys. *J. Mater. Chem. A.* **2014**, *2* (19), 6922-6930.

(18) Liu, W.; Tan, X.; Yin, K.; Liu, H.; Tang, X.; Shi, J.; Zhang, Q.; Uher, C. Convergence of Conduction Bands as a Means of Enhancing Thermoelectric Performance of n-Type $Mg_2Si_{1-x}Sn_x$ Solid Solutions. *Phys. Rev. Lett.* **2012**, *108* (16), 166601.

(19) Deng, Q.; Shi, X. L.; Li, M.; Tan, X. B.; Li, R. H.; Wang, C.; Chen, Y.; Dong, H. L.; Ang, R.; Chen, Z. G. Lattice defect engineering advances n-type PbSe thermoelectrics. *Nat. Commun.* **2025**, *16* (1). 656.

(20) Yin, Z. X.; Zhang, H.; Wang, Y. Q.; Wu, Y.; Xing, Y. B.; Wang, X.; Fang, X. F.; Yu, Y.; Guo, X. Ultrahigh-pressure structural modification in BiCuSeO ceramics: dense dislocations and

exceptional thermoelectric performance. *Adv. Energy Mater.* **2025**, *15* (8), 2403174..
(21) Jiang, B. B.; Liu, X. X.; Wang, Q.; Cui, J.; Jia, B. H.; Zhu, Y. K.; Feng, J. H.; Qiu, Y.; Gu, M.; Ge, Z. H.; et al. Realizing high-efficiency power generation in low-cost PbS-based thermoelectric materials. *Energy Environ. Sci.* **2020**, *13* (2), 579-591.
(22) Byun, S.; Ge, B. Z.; Song, H.; Cho, S. P.; Hong, M. S.; Im, J.; Chung, I. Simultaneously engineering electronic and phonon band structures for high-performance n-type polycrystalline SnSe. *Joule* **2024**, *8* (5), 1520-1538.
(23) Hong, T.; Qin, B. C.; Qin, Y. X.; Bai, S. L.; Wang, Z. Y.; Cao, Q.; Ge, Z. H.; Zhang, X.; Gao, X.; Zhao, L. D. All-SnTe-Based Thermoelectric Power Generation Enabled by Stepwise Optimization of n-Type SnTe. *J. Am. Chem. Soc.* **2024**, *146* (12), 8727-8736.
(24) Wang, D. Z.; Liu, W. D.; Li, M.; Zheng, K.; Hu, H. W.; Yin, L. C.; Wang, Y. F.; Zhu, H.; Shi, X. L.; Yang, X. N.; et al. Hierarchical architectural structures induce high performance in n-type GeTe-based thermoelectrics. *Adv. Funct. Mater.* **2023**, *33* (14), 2213040..
(25) Hu, Y. X.; Bai, S. L.; Wen, Y.; Liu, D. R.; Hong, T.; Liu, S.; Zhan, S. P.; Gao, T.; Chen, P. P.; Li, Y. C.; et al. Stepwise optimization of thermoelectric performance in n-type SnS. *Adv. Funct. Mater.* **2025**, *35* (6). 2414881.
